# Drug-Drug Interactions and the Clinical Tolerability of Colchicine Among Patients With COVID-19: A Secondary Analysis of the COLCORONA Randomized Clinical Trial

**DOI:** 10.1001/jamanetworkopen.2024.31309

**Published:** 2024-09-06

**Authors:** Lama S. Alfehaid, Subrina Farah, Azza Omer, Brittany N. Weber, Omar Alkhezi, Yahya M. K. Tawfik, Amil M. Shah, Peter Libby, Leo F. Buckley

**Affiliations:** 1 Department of Pharmacy, Brigham and Women’s Hospital, Boston, Massachusetts; 2 College of Pharmacy, King Saud bin Abdulaziz University for Health Sciences, Riyadh, Saudi Arabia; 3 Center for Clinical Investigation, Brigham and Women’s Hospital, Boston, Massachusetts; 4 Division of Cardiovascular Medicine, Brigham and Women’s Hospital, Boston, Massachusetts; 5 Department of Pharmacy Practice, College of Pharmacy, Qassim University, Qassim, Saudi Arabia; 6 Department of Clinical Pharmacy, College of Pharmacy, King Saud University, Riyadh, Saudi Arabia; 7 Division of Cardiology, The University of Texas Southwestern Medical Center, Dallas

## Abstract

**Importance:**

Colchicine has many drug-drug interactions with commonly prescribed medications. Only pharmacokinetic studies have provided data on colchicine drug-drug interactions.

**Objective:**

To evaluate the clinical tolerability of colchicine according to the presence or absence of a drug-drug interaction.

**Design, Setting, and Participants:**

A secondary analysis of the COLCORONA trial was performed. The COLCORONA trial was a randomized, double-blind, placebo-controlled trial conducted in Brazil, Canada, Greece, South Africa, Spain, and the US between March 23, 2020, and January 20, 2021. The COLCORONA trial included ambulatory patients with COVID-19 with at least 1 high-risk characteristic and compared the effects of colchicine (0.5 mg twice daily for 3 days, then 0.5 mg daily thereafter) with placebo for 27 days. Data analysis was performed from February 24, 2023, to June 20, 2024.

**Exposure:**

In this secondary analysis, baseline medications that had interactions with colchicine were identified using a previously published expert classification.

**Main Outcomes and Measures:**

The primary outcome for this analysis was the composite of serious and nonserious treatment-related and treatment-unrelated gastrointestinal adverse events. The secondary outcomes were other adverse events and the composite of death or hospital admission due to COVID-19 infection. Logistic regression models adjusted for age, sex, estimated glomerular filtration rate, diabetes, heart failure, and myocardial infarction were assessed for effect modification of the association between the randomization arm and the outcomes of interest by drug-drug interaction status.

**Results:**

The cohort included 2205 participants in the colchicine arm and 2227 in the placebo arm (median age, 54 [IQR, 47-61] years; 2389 [54%] women). The most common colchicine drug-drug interactions were rosuvastatin (12%) and atorvastatin (10%). In fully adjusted models, the odds of any gastrointestinal adverse event were 1.80 (95% CI, 1.51-2.15) times higher in the colchicine arm than the placebo arm among people without a drug-drug interaction and 1.68 (95% CI, 1.24-2.26) times higher in the colchicine arm than the placebo arm among people with a drug-drug interaction (*P* = .69 for interaction). Drug-drug interaction status did not significantly modify the effect of colchicine on the composite of COVID-19 hospitalization or death (odds ratio, 0.91; 95% CI, 0.59-1.40 for drug-drug interaction and 0.84; 95% CI, 0.60-1.19 for no drug-drug interaction; *P* = .80 for interaction).

**Conclusions and Relevance:**

In this secondary analysis of the COLCORONA trial, operational classification of drug interactions system class 3 or 4 drug-drug interactions did not appear to significantly increase the risk of colchicine-related adverse effects.

**Trial Registration:**

ClinicalTrials.gov Identifier: NCT04322682

## Key Points

**Question:**

Are drug-drug interactions associated with the clinical safety and efficacy of colchicine in people with COVID-19?

**Findings:**

In this secondary analysis of the COLCORONA trial including 4432 patients, interactions with statins and calcium channel blockers did not modify the association between randomization to colchicine vs placebo and the risk of any safety end point. Findings were similar for efficacy end points.

**Meaning:**

The findings of this study suggest that drug-drug interaction-associated changes in colchicine pharmacokinetics do not translate into clinically significant changes in its safety and efficacy profile.

## Teaser

This secondary analysis of a randomized clinical trial examines gastrointestinal adverse events due to interactions between colchicine and other medications in individuals with COVID-19.

## Introduction

Colchicine, an anti-inflammatory agent, can aid management of a wide range of inflammatory diseases, including gout, familial Mediterranean fever, pericarditis, and atherosclerotic cardiovascular disease.^1^ Colchicine can cause dose-limiting diarrhea, nausea, vomiting, gastrointestinal pain, and muscle pain.^1^ Concern regarding rare but more serious adverse effects attributed to colchicine, such as neutropenia and thrombocytopenia, has arisen primarily from case reports and case series.^1^

The cytochrome P450 3A4 (CYP3A4) enzyme transforms colchicine into inactive metabolites, while the xenobiotic transporter P-glycoprotein (P-gp) extrudes colchicine from epithelial cells in the gastrointestinal tract, liver, and kidney.^2^ Drugs that inhibit CYP3A4 or P-gp activity can increase colchicine exposure.^2^ Clinicians may frequently encounter colchicine drug-drug interactions, such as those involving statins and nondihydropyridine calcium channel blockers. Evidence to guide the management of these drug-drug interactions, however, includes only case reports and pharmacokinetic studies.^2^ Due to the lack of data on the association between colchicine plasma concentrations and its pharmacodynamic or clinical effects, pharmacokinetic data provide limited insight into clinical decision-making. Moreover, patients and clinicians have the greatest interest in the effects of drug-drug interactions on the occurrence of clinical events, rather than changes in pharmacokinetic parameters.

We conducted a post hoc analysis of the COLCORONA trial, which randomized 4488 ambulatory individuals with COVID-19 to colchicine or placebo for 30 days, to examine whether the presence of a drug-drug interaction at baseline modified the effects of colchicine on the clinical end points of gastrointestinal adverse events and the composite of hospitalization for COVID-19 or death.

## Methods

### Study Design

This study was a post hoc analysis of the international, randomized, double-blind, placebo-controlled COLCORONA trial that was conducted in Brazil, Canada, Greece, South Africa, Spain, and the US.^3^ The COLCORONA trial compared the efficacy and safety of colchicine, 0.5 mg, twice daily for 3 days followed by once daily for 27 days and placebo in ambulatory, high-risk people with COVID-19 over a 30-day follow-up period. All COLCORONA participants provided written informed consent. The COLCORONA trial began on March 23, 2020, and ended on January 20, 2021. The present analysis was determined to be not human participant research by the Mass General Brigham Institutional Review Board. The COLCORONA trial was approved by the institutional review board at each participating center. Patients or the public were not involved in the design, conduct, reporting, or dissemination plans of our research. We used the Consolidated Standards of Reporting Trials (CONSORT) reporting guideline when drafting our report.^4^ The COLCORONA trial protocol is available in Supplement 1 and the statistical analysis plan is available in Supplement 2.

### Study Participants

This post hoc analysis included all COLCORONA participants in the intention-to-treat cohort who had available covariate data. The overall COLCORONA cohort included people who were at least aged 40 years, had COVID-19 infection, and had at least 1 of the following high-risk criteria: aged 70 years or older, diabetes, systolic blood pressure of at least 150 mm Hg, respiratory disease, heart failure, coronary disease, temperature of 38.4 °C or higher within the last 48 hours, dyspnea, bicytopenia, pancytopenia, or high neutrophil count with low lymphocyte count.^3^ Major exclusion criteria for the overall COLCORONA cohort included a history of an allergic reaction or major sensitivity to colchicine, a current indication for colchicine, inflammatory bowel disease, chronic diarrhea, chronic malabsorption, progressive neuromuscular disease, Modification of Diet in Renal Disease estimated glomerular filtration rate (eGFR) less than 30 mL/min/1.73 m^2^, and a history of cirrhosis, chronic active hepatitis, severe hepatic disease, or cancer (if undergoing chemotherapy). In addition, the study protocol prohibited concomitant use of erythromycin, clarithromycin, cyclosporine, and verapamil, or consumption of grapefruit juice. The enrollment of participants taking other moderate or strong CYP3A4 inhibitors or P-gp inhibitors or substrates was allowable on a case-by-case basis. Use of other medications was allowed during the trial if the regimens were stabilized prior to study entry and remained stable throughout the study.

### Drug-Drug Interaction Classification

All medications used routinely at the time of randomization were recorded in the case report form. Recorded medication names were mapped to the World Health Organization Drug Dictionary by medical coders.^5^ We defined a drug-drug interaction as the concomitant use of any medication previously classified as having an Operational Classification (ORCA) of Drug Interactions^6^ class 1 (contraindicated), class 2 (provisionally contraindicated), class 3 (conditional use), or class 4 (minimal risk) interaction with colchicine by an expert panel.^2^ We excluded drug-drug interactions that involved CYP3A4 inducers due to the small numbers of these interactions (n = 6). We considered alternative drug-drug interaction classification systems, but all systems agreed well except 1, which classified most ORCA drug-drug interactions as not significant (eTable 1 in Supplement 3).

### Outcomes

The primary outcome of interest was any gastrointestinal adverse event. The safety outcomes of interest were the composite of serious and nonserious, treatment-related and treatment-unrelated gastrointestinal adverse events, and overall adverse events. The efficacy outcome of interest was the composite of death or hospital admission due to COVID-19 infection (COLCORONA primary end point). Outcomes were assessed for 30 days after randomization. Outcome ascertainment occurred via telephone calls at 15 and 30 days from randomization.

### Statistical Analysis

Data analysis was conducted from February 24, 2023, to June 20, 2024. The sample size was based on the number of participants with available data. Participant characteristics were summarized as count (percentage) or median (IQR) as appropriate. The effect of colchicine on safety and efficacy outcomes, accounting for the interaction between treatment arm and drug-drug interaction status, was assessed using logistic regression models. We adjusted for demographic characteristics and then demographic characteristics plus medical history in separate models to assess the effect of different covariate groups on the associations of interest. Covariates were selected based on prior knowledge, clinical experience, and data availability. Model 1 included age, sex, eGFR, and the interaction between the randomization arm and drug-drug interaction status. The fully adjusted model (model 2) included age, sex, eGFR, diabetes, heart failure, myocardial infarction, and the interaction between treatment arm and drug-drug interaction status. The primary test of interest was the interaction between randomization arm and drug-drug interaction status, assessed using a multiplicative interaction term. Data were analyzed using Stata, version 17.0 (StataCorp LLC). A 2-sided value of *P* < .05 was considered statistically significant.

## Results

### Participant Characteristics

The study cohort included 4432 participants (2389 [54%] women, 2043 [46%] men) with a median age of 54 (IQR, 47-61) years (eFigure in Supplement 3). The median eGFR was 96 (IQR, 84-105) mL/min/1.73 m^2^, and 3% of participants had an eGFR less than 60 mL/min/1.73 m^2^. Diabetes (20%) and hypertension (36%) were the most common comorbidities.

In both the placebo and colchicine arms, participants receiving at least 1 medication that interacts with colchicine were older and more likely to have diabetes, hypertension, heart failure, or a prior myocardial infarction (Table 1). The median eGFR was lower among participants receiving a medication that interacts with colchicine than those not receiving such drugs (Table 2).

**Table 1.** Baseline Characteristics

Characteristic	No. (%)			
	Colchicine		Placebo	
	No DDI (n = 1589)	ORCA 1-4 DDI (n = 616)^a^	No DDI (n = 1624)	ORCA 1-4 DDI (n = 603)^a^
Age, median (IQR), y	51 (45-58)	59 (52-65)	52 (45-58)	60 (54-67)
Women	959 (60)	262 (43)	941 (58)	227 (38)
Men	630 (40)	354 (57)	683 (42)	376 (62)
Diabetes	158 (10)	276 (45)	157 (10)	283 (47)
Hypertension	390 (25)	379 (62)	430 (27)	400 (66)
Prior myocardial infraction	12 (<1)	51 (8)	9 (<1)	59 (10)
Prior heart failure	7 (<1)	16 (3)	5 (<1)	12 (2)
History of respiratory disease	344 (22)	142 (23)	392 (24)	125 (21)
eGFR, mL/min/1.73 m^2^, median (IQR)	98 (85-107)	93 (81-102)	98 (85-107)	91 (77-101)
eGFR <60 mL/min/1.73 m^2^	36 (2)	28 (5)	52 (3)	37 (6)

Abbreviations: DDI, drug-drug interaction; eGFR, estimated glomerular filtration rate (Chronic Kidney Disease Epidemiology Collaboration 2021); ORCA, Operational Classification of Drug Interactions.

^a^ ORCA classification: class 1 (contraindicated), class 2 (provisionally contraindicated), class 3 (conditional use), and class 4 (minimal risk).

**Table 2.** Drug-Drug Interactions at Randomization According to Treatment Arm

Drug-drug interaction class^a^	No. (%)	
	Colchicine (n = 2205)	Placebo (n = 2227)
ORCA class 1 inhibitors, No.	0	0
ORCA class 2 inhibitors, No.	20 (<1)	22 (<1)
ORCA class 3 inhibitors, No.		
1	288 (13)	328 (15)
2	15 (<1)	4 (<1)
3	1 (<1)	0
ORCA class 4 inhibitors		
1	309 (14)	280 (13)
2	2 (<1)	3 (<1)

Abbreviation: ORCA, Operational Classification of Drug Interactions.

^a^ ORCA classification: class 1 (contraindicated), class 2 (provisionally contraindicated), class 3 (conditional use), and class 4 (minimal risk).

The number of participants across both treatment arms who reported receiving medications at randomization that interact with colchicine was 42 (1%) for medications with an ORCA class 2 interaction, 636 (14%) for medications with a class 3 interaction, and 594 (13%) for medications with a class 4 interaction (Table 2). None of the participants reported receiving medication with an ORCA class 1 colchicine interaction. The most common interacting medications reported were rosuvastatin (12% of all participants) and atorvastatin (10% of all participants) (eTable 2 in Supplement 3).

### Safety and Efficacy of Colchicine vs Placebo According to Drug-Drug Interaction Status

Adverse event rates generally were higher in the colchicine arm than in the placebo arm, regardless of whether participants were receiving a drug that interacts with colchicine at baseline (Table 3). Any gastrointestinal adverse event occurred in 134 (22%) participants with and 391 (25%) of those without a drug-drug interaction in the colchicine arm compared with 84 (14%) participants with and 249 (15%) of those without in the placebo arm. After adjustment for age, sex, eGFR, diabetes, heart failure, and myocardial infarction (model 2), the risk of any gastrointestinal adverse event was 1.80 (odds ratio [OR], 1.80; 95% CI, 1.51-2.15) times higher in the colchicine arm than the placebo arm among people without a drug-drug interaction and 1.68 (OR, 1.68; 95% CI, 1.24-2.26) times higher in the colchicine arm than the placebo arm among people with a drug-drug interaction (*P* = .69 for interaction). In the fully adjusted model (model 2), the effect of colchicine compared with placebo on serious gastrointestinal adverse events and treatment-related gastrointestinal adverse events did not differ significantly between participants receiving and not receiving a drug that interacts with colchicine (Table 3). Similar results were applied to overall adverse events, overall serious adverse events, and overall treatment-related adverse events (Table 3, Figure).

**Table 3.** Effect of Colchicine on Gastrointestinal Adverse Events According to Presence of an ORCA Class 1-3 Drug-Drug Interaction at Baseline^a^

DDI status	**AEs, No. (%)**		Unadjusted		Model 1^b^		Model 2^c^	
	Colchicine	Placebo	OR (95% CI)	*P* value for interaction	OR (95% CI)	*P* value for interaction	OR (95% CI)	*P* value for interaction
**Any GI AE**								
DDI	134 (22)	84 (14)	1.72 (1.27-2.32)	.79	1.68 (1.24-2.26)	.71	1.68 (1.24-2.26)	.69
No DDI	391 (25)	249 (15)	1.80 (1.51-2.15)		1.79 (1.50-2.14)		1.80 (1.51-2.15)	
**Serious GI AEs**								
DDI	1 (<1)	1 (<1)	0.98 (0.06-15.69)	.66	0.94 (0.06-15.10)	.65	0.93 (0.06-14.92)	.64
No DDI	4 (<1)	2 (<1)	2.05 (0.37-11.19)		2.01 (0.37-10.99)		2.02 (0.37-11.09)	
**Treatment-related GI AE**								
DDI	130 (21)	80 (13)	1.75 (1.29-2.37)	.72	1.71 (1.26-2.32)	.65	1.71 (1.26-2.32)	.64
No DDI	384 (24)	237 (15)	1.86 (1.56-2.23)		1.85 (1.55-2.22)		1.86 (1.55-2.23)	
**Any AE**								
DDI	174 (28)	129 (21)	1.45 (1.11-1.88)	.91	1.47 (1.25-1.73)	.90	1.44 (1.11-1.87)	.86
No DDI	461 (29)	353 (22)	1.47 (1.25-1.73)		1.44 (1.11-1.87)		1.48 (1.26-1.74)	
**Serious AE**								
DDI	44 (7)	49 (8)	0.87 (0.56-1.33)	.64	0.93 (0.61-1.42)	.52	0.80 (0.58-1.10)	.58
No DDI	69 (4)	91 (6)	0.76 (0.55-1.05)		0.78 (0.56-1.08)		0.93 (0.61-1.43)	
**Treatment-related AE**								
DDI	135 (22)	85 (14)	1.71 (1.27-2.31)	.97	1.67 (1.24-2.25)	.89	1.67 (1.24-2.26)	.89
No DDI	398 (25)	264 (16)	1.72 (1.45-2.05)		1.71 (1.44-2.03)		1.71 (1.44-2.04)	
**COVID-19 hospitalization or death**								
DDI	42 (7)	48 (8)	0.85 (0.55-1.30)	.86	0.90 (0.58-1.39)	.74	0.91 (0.59-1.40)	.80
No DDI	62 (4)	78 (5)	0.80 (0.57-1.13)		0.82 (0.58-1.16)		0.84 (0.60-1.19)	

Abbreviations: AE, adverse event; DDI, drug-drug interaction; GI, gastrointestinal; OR, odds ratio; ORCA, Operational Classification of Drug Interactions.

^a^ ORCA classification: class 1 (contraindicated), class 2 (provisionally contraindicated), class 3 (conditional use), and class 4 (minimal risk).

^b^ Model 1 includes adjustments for age, sex, estimated glomerular filtration rate (eGFR), and the interaction between the treatment arm and drug-drug interaction status.

^c^ Model 2 includes adjustments for age, sex, eGFR, diabetes, heart failure, myocardial infarction, and the interaction between the treatment arm and drug-drug interaction status.

**Figure.**
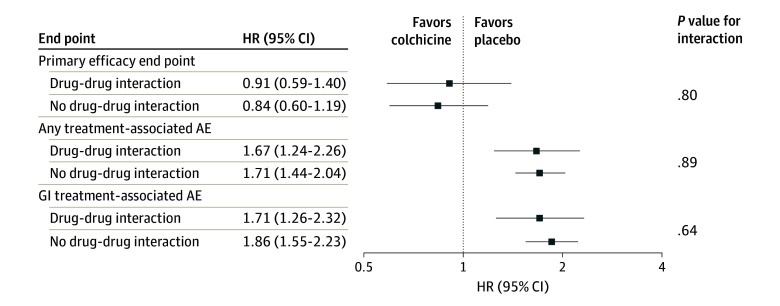
Effect of Colchicine vs Placebo on Efficacy and Safety End Points in People With COVID-19 According to the Presence or Absence of a Drug-Drug Interaction at Baseline AE indicates adverse event; GI, gastrointestinal; and HR, hazard ratio.

The COLCORONA primary efficacy end point of COVID-19 hospitalization or death occurred in 42 (7%) participants with and 62 (4%) of those without a drug-drug interaction in the colchicine arm and 48 (8%) participants with and 78 (5%) of those without a drug-drug interaction in the placebo arm. Drug-drug interaction status did not significantly modify the effect of colchicine on the composite of COVID-19 hospitalization or death (Table 3).

## Discussion

In this post hoc analysis of the randomized, double-blind, placebo-controlled COLCORONA trial, the presence of a drug-drug interaction leading to higher colchicine exposure at baseline was not associated with an increase in the risk of overall or gastrointestinal adverse events in the colchicine arm. Most drug-drug interactions were classified as ORCA class 3 or 4, and individuals at the highest risk of colchicine-related adverse events, such as those with chronic kidney disease or gastrointestinal disorders, were excluded from the COLCORONA trial. Thus, the findings of this analysis suggest that low-dose colchicine may be continued despite certain drug-drug interactions in carefully selected individuals who receive close follow-up.

Colchicine exerts anti-inflammatory effects by inhibiting microtubule polymerization within neutrophils and other professional and nonprofessional immune cells.^7,8^ The adverse gastrointestinal effects of colchicine have been known for millennia, but the tolerability and efficacy of low-dose colchicine regimens were established first in 2010.^1,9^ The pharmacokinetic effects of colchicine drug-drug interactions of varying severity have been studied,^2^ but the clinical consequences remain unclear. Our study provides evidence suggesting that colchicine can be used safely in the presence of ORCA class 3 or 4 drug-drug interactions in individuals without other risk factors who receive close follow-up.

The most frequent drug-drug interactions in this study involved atorvastatin and rosuvastatin, statins that provide high-intensity decreases in low-density lipoprotein cholesterol levels. Colchicine interactions with statins have particular importance given the prevalence of statin use and between-statin differences in pharmacokinetic metabolism and risk of muscle-related adverse effects. Atorvastatin metabolism and excretion occur through CYP3A4 and P-gp, whereas rosuvastatin relies primarily on CYP2C9 and organic anion transporter family member 1B1. Atorvastatin may increase total colchicine exposure by approximately 24%, whereas rosuvastatin should have no effect on colchicine exposure.^10^ Case reports describe colchicine adverse effects among people receiving concomitant atorvastatin and rosuvastatin.^11^ In contrast, our analysis, which included participants randomly assigned to colchicine and placebo and used prospective assessment of concomitant medication and adverse events, suggests that the modestly increased colchicine exposure with concomitant atorvastatin use has little clinical relevance. In addition, our results agree with the tolerability of colchicine and rosuvastatin in 2 clinical trials comparing colchicine and rosuvastatin vs standard of care in COVID-19, in which adverse events occurred infrequently in the colchicine and rosuvastatin arm.^12,13^ The management of other drug-drug interactions, such as colchicine and simvastatin,^14^ should be evaluated on a case-by-case basis due to the small numbers in our analysis.

The colchicine dosing regimen may alter the effect of drug-drug interactions on its tolerability. After an initial 3-day loading dose, COLCORONA participants received a low-dose colchicine regimen of 0.5 mg daily.^3^ Colchicine, 0.5 mg, daily reduces cardiovascular risk in coronary artery disease with a strong safety and tolerability profile.^15,16^ In contrast, higher colchicine doses are used in pericarditis and familial Mediterranean fever.^17^ Higher colchicine doses may accentuate the effect of drug-drug interactions on colchicine tolerability.

Our study highlights the challenges with evaluation of drug-related adverse effects and drug-drug interactions. Drug-drug interaction studies predominantly focus on pharmacokinetic end points, such as maximal concentration and area under the curve, but the associations between pharmacokinetic effects and either pharmacodynamic effects or clinical events have not been established for most drugs. Case reports and case series, which compose most of the remaining data on drug-drug interactions, can identify novel, hypothesis-generating findings, but have many limitations compared with higher levels of evidence, such as cohort studies and randomized clinical trials. Even randomized clinical trials have limitations, such as the exclusion of individuals at highest risk for a drug adverse effect, protocol-mandated avoidance of drug-drug interactions based on pharmacokinetic studies, and small numbers of rare events. The ideal body of evidence to inform drug-drug interaction management should include pharmacokinetic studies, which provide mechanistic insight, post hoc analyses of randomized clinical trials (where available), and studies of routinely collected data, which include higher-risk individuals and drug-drug interactions of medications more frequently used.

### Limitations

This study has certain limitations. First, this secondary analysis focuses on medications used by participants at baseline, which were categorized according to their potential for and severity of drug interactions. Medications that have interactions with colchicine but were not used or were used in a small proportion of study participants were not analyzed. Additionally, the findings of this study do not provide information on rare adverse events, such as rhabdomyolysis, which usually occur months after starting drug therapy. Furthermore, all study participants had confirmed SARS-CoV-2 infection, which may have increased their susceptibility to adverse reactions associated with colchicine use.

## Conclusions

In this secondary analysis of a randomized clinical trial, in carefully selected individuals under close monitoring, ORCA class 3 or 4 drug-drug interactions did not appear to significantly increase the risk of colchicine-related gastrointestinal adverse effects. These data may alleviate concerns over the safety of colchicine in patients receiving an interacting drug.

## Notes

Supplement 1.
Trial Protocol

Supplement 2.
Statistical Analysis Plan

Supplement 3. **eFigure.** CONSORT Flow Diagram **eTable 1.** Comparison of Drug-Drug Interaction Classifications **eTable 2.** Use of Drugs That Interact With Colchicine at Baseline

Supplement 4.
Data Sharing Statement
