# Supplementary material for: Drug-Drug Interactions and the Clinical Tolerability of Colchicine Among Patients With COVID-19: A Secondary Analysis of the COLCORONA Randomized Clinical Trial
Source: JAMA Netw Open. 2024 Sep 6;7(9):e2431309. doi: 10.1001/jamanetworkopen.2024.31309 (PMC11380098; doi:10.1001/jamanetworkopen.2024.31309)
Supplement: Supplement 1. — Trial Protocol [file jamanetwopen-e2431309-s001.pdf]

CLINICAL RESEARCH PROTOCOL

Protocol # MHIPS-2020-001

COLCHICINE CORONAVIRUS SARS-CoV2 TRIAL (COLCORONA)

March 20th, 2020

SPONSOR

MONTREAL HEART INSTITUTE

CONFIDENTIALITY

The Montreal Health Innovations Coordinating Center (MHICC), a division of the Montreal Heart Institute (MHI), considers the information contained in this document to be proprietary and/or confidential. It is for the use of the investigational team, and the corresponding applications to institutional review boards (IRBs) and independent ethics committees (IECs) in support of a clinical trial. Accordingly, please do not copy, use, distribute, or disclose to others without the written prior approval of MHICC or their designated representatives.

MHICC signatures for:

Protocol # MHIPS-2020-001
COLCHICINE CORONARYVIRUS SARS-COV2 TRIAL (COLCORONA)

Andreas Orfanos, MBBCh, FFPM, MBA<br>Director, MHICC	(Signature)	20MAR2020<br>(Date)
Marie-Claude Guertin, Ph.D.<br>Head, Biostatistics Department, MHICC	(Signature)	(Date)
Jean-Claude Tardif, MD<br>Director, MHI Research Center<br>Principal Investigator	(Signature)	(Date)

MHICC signatures for:

Protocol # MHIPS-2020-001
COLCHICINE CORONARYVIRUS SARS-COV2 TRIAL (COLCORONA)

Andreas Orfanos, MBBCh, FFPM, MBA<br>Director, MHICC	(Signature)	(Date)
Marie-Claude Guertin, Ph.D.<br>Head, Biostatistics Department, MHICC	[signature]<br>(Signature)	22 MAR 2020<br>(Date)
Jean-Claude Tardif, MD<br>Director, MHI Research Center<br>Principal Investigator	(Signature)	(Date)

MHICC signatures for:

Protocol # MHIPS-2020-001
COLCHICINE CORONARYVIRUS SARS-COV2 TRIAL (COLCORONA)

Andreas Orfanos, MBBCh, FFPM, MBA
Director, MHICC

____
(Signature) (Date)

Marie-Claude Guertin, Ph.D.
Head, Biostatistics Department, MHICC

____
(Signature) (Date)

Jean-Claude Tardif, MD
Director, MHI Research Center
Principal Investigator

2020-MAR-22
____
(Signature) (Date)

Table of Contents

INVESTIGATOR'S SIGNATURE 5
SYNOPSIS 6
TIME PERIOD AND NUMBER OF PATIENTS: 10
1 INTRODUCTION 11
1.1 Background 11
1.2 Background on colchicine 12
1.3 Colchicine Dosage 13
2 STUDY OBJECTIVES 13
3 STUDY DESIGN 13
3.1 Study Schedule 14
3.2 Enrollment 14
3.3 Screening Evaluations and Randomization 14
3.4 Active Treatment Period 15
Table 1 Timetable of Visits and Procedures 16
4 STUDY POPULATION 17
4.1 Source and Number of Patients 17
4.2 Patient Selection Criteria 17
4.2.1 Inclusion Criteria: 17
4.2.2 Exclusion Criteria: 17
4.3 Prohibited, Allowable and Concurrent Medications 18
5 STUDY METHODOLOGY 18
5.1 Efficacy Outcomes 18
5.2 Safety Monitoring 19
5.2.1 Adverse Event Reporting 19
5.2.2 Other Safety Monitoring 21
5.3 Withdrawal of Patients from the Study 21
5.4 Study Completion 22
6 STUDY MEDICATION 22
6.1 Medication Dispensing 22
6.2 Dosage Regimen 22
7 DATA COLLECTION 23
8 DATA ANALYSIS AND STATISTICAL CONSIDERATIONS 23
8.1 Statistical Power and Sample Size Considerations 23
8.2 Analysis population 23
8.2.1 Intent to Treat (ITT) population 23
8.2.2 Safety population 23
8.3 Data analysis 23
8.3.1 Analysis of efficacy outcomes 23
8.3.2 Analysis of safety outcomes 24
8.3.3 Interim analysis 24

9 STUDY COORDINATION 24
REFERENCES 25
LIST OF APPENDICES 28
Appendix A Detailed Study Safety Parameters 29
Appendix B PMS-Colchicine 0.6mg Product Monograph..31
Appendix C Administrative Procedures for the Reporting of Adverse Events.....................49
1 ADVERSE EVENTS DURING THE TRIAL...49
1.1 Pre-existing condition 49
1.2 Adverse Event (AE) 49
1.3 Related Adverse Event...49
1.4 Serious Adverse Event (SAE)..50
1.5 Life-Threatening Adverse Event 50
1.6 Unexpected Adverse Event 50
1.7 Clinical Laboratory Adverse Event..51
1.8 Treatment-Emergent Signs and Symptoms (TESS)...51
1.9 Post-treatment Adverse Event 51
2 HANDLING OF ADVERSE EVENTS..51
2.1 Treatment-Emergent Signs and Symptoms 51
2.2 Serious Adverse Events 51
2.3 Intensity..51
2.4 Relationship to Study Drug – Physician's Assessment 51
2.5 Clinical Outcome 51
3 CAPTURING AVDERSE EVENTS...52
3.1 Pre-Existing Condition 52
3.2 Lack of Efficacy of Study Medication 52
3.3 Clinical Laboratory Adverse Event...52
3.4 Hospitalization or Surgery/Procedure 52
3.5 Death...52
4 REPORTING TO THE SPONSOR 52
4.1 Immediately Reportable Adverse Events 53
4.2 Other Adverse Events...53
4.3 Follow-Up Period 53
Appendix D Other Administrative and Regulatory Procedures...54
Appendix E Declaration of Helsinki 59

INVESTIGATOR'S SIGNATURE

I have read the protocol and agree to conduct this trial in accordance with all stipulations of the protocol, with applicable laws and regulations and in accordance with the ethical principles outlined in the Declaration of Helsinki.

By signing below, I hereby declare that I am not debarred, disqualified, or otherwise restricted by any agency from conducting any research studies.

Principal Investigator	Signature	Date

Country:

PROTOCOL MHIPS
SYNOPSIS
TITLE: COLCHICINE CORONAVIRUS SARS-CoV2 TRIAL (COLCORONA)

INDICATION: Reduction of complications in patients with COVID-19 infection.

OBJECTIVES: The primary objective of this study is to determine whether short-term treatment with colchicine reduces the rate of death and lung complications related to COVID-19. The secondary objective is to determine the safety of treatment with colchicine in this patient population. The exploratory objective is to evaluate links between soluble and genetic biomarkers and treatment effects.

PATIENT POPULATION: Males and females, at least 40 years of age, who have been diagnosed with a COVID-19 infection and have at least one high-risk criterion.

Inclusion Criteria:

- Males and females, at least 40 years of age, capable and willing to provide informed consent;
- Patient must have received a diagnosis of COVID-19 infection within the last 24 hours;
- Outpatient setting (not currently hospitalized or under immediate consideration for hospitalization);
- Patient must possess at least one of the following high-risk criteria: 70 years or more of age, diabetes mellitus, uncontrolled hypertension (systolic blood pressure ≥150 mm Hg), known respiratory disease (including asthma or chronic obstructive pulmonary disease), known heart failure, known coronary disease, fever of ≥38.4°C within the last 48 hours, dyspnea at the time of presentation, bicytopenia, pancytopenia, or the combination of high neutrophil count and low lymphocyte count;
- Female patient is either not of childbearing potential, defined as postmenopausal for at least 1 year or surgically sterile, or is of childbearing potential and practicing at least one method of contraception and preferably two complementary forms of contraception including a barrier method (e.g. male or female condoms, spermicides, sponges, foams, jellies, diaphragm, intrauterine device (IUD)) throughout the study and for 30 days after study completion;
- Patient must be able and willing to comply with the requirements of this study protocol.

Exclusion Criteria:

- Patient currently hospitalized or under immediate consideration for hospitalization;
- Patient currently in shock or with hemodynamic instability;

- Patient with inflammatory bowel disease (Crohn's disease or ulcerative colitis), chronic diarrhea or malabsorption;
- Patient with pre-existent progressive neuromuscular disease;
- Estimate Glomerular filtration rate (eGFR) using the MDRD equation for all subjects being considered for enrollment, with a cut-off of < 30 mL/m in/1.73m^2.
- Patient with a history of cirrhosis, chronic active hepatitis or severe hepatic disease;
- Female patient who is pregnant, or breast-feeding or is considering becoming pregnant during the study or for 6 months after the last dose of study medication;
- Patient currently taking colchicine for other indications (mainly chronic indications represented by Familial Mediterranean Fever or gout). There is no wash-out period required for patients who have been treated with colchicine and stopped treatment prior to enrolment;
- Patient with a history of an allergic reaction or significant sensitivity to colchicine;
- Patient undergoing chemotherapy for cancer;
- Patient is considered by the investigator, for any reason, to be an unsuitable candidate for the study.

STUDY DESIGN: This will be a randomized, double-blind, placebo-controlled, multi-center study. Following signature of the informed consent form, approximately 6000 subjects meeting all inclusion and no exclusion criteria will be randomized to receive either colchicine or placebo (1:1 allocation ratio) for 30 days. Follow-up phone or video assessments will occur at 15 and 30 days following randomization for evaluation of the occurrence of any trial endpoints or other adverse events.

OUTCOMES: The primary endpoint will be the composite of death or the need for hospitalization due to COVID-19 infection in the 30 days following randomization. The secondary endpoints will consist of the components of the composite primary endpoint; and the need for mechanical ventilation in the 30 days following randomization. Exploratory endpoints will include associations between both biomarkers and viral load (stored samples) and treatment effects.

STATISTICAL RATIONALE AND ANALYSIS: All main analyses in COLCORONA will be conducted on an intention-to-treat basis. A sample size of approximately 6000 randomized patients with 3000 patients in each treatment group will be required to detect a 25% risk reduction with colchicine with a power of 80% given a primary endpoint event rate of 7% in the placebo group and a two-sided test at the 0.05 significance level.

A fully independent 5-member Data and Safety Monitoring Board (DSMB) will be established and will review unblinded safety data as detailed in the DSMB charter. An interim analysis is planned after approximately 50% of randomized patients have completed 30 days of follow-up.

The DSMB charter will pre-specify the methods of interim efficacy analyses and the rules for early study termination, approved by all board members. The stopping rules for efficacy and futility will be based on the O'Brien-Fleming alpha-spending function or on a similar conservative approach so that the impact on the final alpha level will be negligible. The DSMB will have the option of recommending early study termination because of overwhelming efficacy, early termination for futility, or continuation of the trial as planned.

The primary analysis of efficacy will be based on the intent-to-treat principle. The primary endpoint will be compared between the two treatment groups using a chi-square test. Secondary endpoints will be analyzed similarly.

ANTICIPATED TOTAL NUMBER OF RANDOMIZED PATIENTS: Approximately 6000 patients in total will be randomized in this study.

PHASE: 3

STUDY LOCATION: Canada

GLOSSARY

ACE	-	Angiotensin Converting Enzyme
ACE2	-	Angiotensin Converting Enzyme 2
AE	-	Adverse event
ALT	-	Alanine aminotransferase
AST	-	Aspartate aminotransferase
BUN	-	Blood urea nitrogen
eCRF	-	electronic Case Report Form
CPK	-	Creatine phosphokinase
CYP3A4	-	cytochrome P450 3A4
DSMB	-	Data and Safety Monitoring Board
EC	-	Ethics Committee
GGT	-	Gamma-glutamyl transferase
hs-CRP	-	High sensitivity C-reactive protein
ICF		Informed Consent Form
ICH	-	International Council for Harmonisation
IL	-	Interleukin
IMPI	-	Investigational Medicinal Product Information
ITT	-	Intent-to-treat
IUD	-	Intrauterine device
IRB	-	Institutional Review Board
LDH	-	Lactate dehydrogenase
MDR	-	Medication dispensing record
MHI	-	Montreal Heart Institute
MHICC	-	Montreal Health Innovations Coordinating Center
P-gp	-	P-glycoprotein
PO (po)	-	Per os
PRN	-	pro re nata (as necessary)
RAAS	-	Renin-angiotensin-aldosterone system
RBC	-	Red blood cells
SAE	-	Serious adverse event
SOPs	-	Standard operating procedures
SUSAR	-	Suspected Unexpected Serious Adverse Reaction
TESS	-	Treatment-Emergent Signs and Symptoms
WMA	-	World Medical Association

TIME PERIOD AND NUMBER OF PATIENTS:

A. Anticipated Starting Date of Study: 1Q20
B. Anticipated Completion Date: 3Q20
C. Anticipated Number of Patients per Site: Centralized recruitment and follow-up
D. Anticipated Number of Sites: Centralized recruitment and follow-up

Generic Name	Strength and Dosage Form	Therapeutic Classification
Colchicine	0.5 mg tablet	Immuno-modulatory
	Placebo to match 0.5 mg tablet	NA

1 INTRODUCTION

1.1 Background

COVID-19 disease

COVID-19 is due to an infection by the beta-coronavirus SARS-CoV2 (1). The outbreak of COVID-19 disease started in December 2019 in Wuhan, Hubei Province, China (1,2), has spread to other parts of China and Asia, and is now a pandemic that has reached Europe and North America. The number of confirmed cases has reached 170,000, including >6500 deaths, as of March 16, 2020. Patients initially present with fever with or without respiratory symptoms, but a large number of patients later develop various degrees of pulmonary abnormalities on chest imaging (3). Although the vast majority of patients only have a common, mild form of illness, approximately 15% of the patients fall into the severe group, with requirement of assisted ventilation and oxygenation (3). These patients suffer from acute respiratory distress syndrome. Pathologic findings reveal edema and prominent proteinaceous exudates, vascular congestion, and inflammatory clusters with fibrinoid material and multinucleated giant cells (4).

This single-strand RNA virus depends on angiotensin converting enzyme 2 (ACE2) for its entry into cells (5). ACE2 is primarily produced in Clara cells and type II alveolar epithelial cells (6).

Host response to viral infection

Accumulating evidence suggests that a subgroup of patients with severe COVID-19 might have a cytokine storm syndrome (7). Identification and treatment of this dysregulated (exaggerated) inflammatory response using existing, approved therapies with proven safety profiles has been advocated to address the immediate need to reduce the rising mortality (7,8).

A characteristic common to both the 1918 Spanish flu pandemic and the current COVID-19 pandemic is that children are largely protected from severe complications of the infection (9). Unlike macrophages from adults, ex vivo responses of children macrophages to endotoxin and interleukin-1 are dominated by an interleukin-10-dependent anti-inflammatory pattern (10, 11). Recent research has shown that DNA methylation stably reduces the expression of IL-10 in Th1 cells (12). In the absence of IL-10 signaling, macrophages accumulate damaged mitochondria in a mouse model of colitis and in patients with inflammatory bowel disease, resulting in dysregulated activation of the NLRP3 inflammasome and production of IL-1β (13). Thus, these observations suggest that the protection against complications of pandemic influenza and COVID-19 in children might be explained by their ability to rapidly mount an IL-10-dependent anti-inflammatory response. Therefore, we hypothesize that targeting the NLRP3 inflammasome

may reduce COVID-19-related complications in adults at risk or with evidence of cytokine activation.

1.2 Background on colchicine

Colchicine is an inexpensive, yet potent, anti-inflammatory drug that is approved for acute use in patients with gout and chronic use in patients with Familial Mediterranean Fever, at doses between 0.3 mg and 2.4 mg/day (see product monograph in Appendix B). The active compound, initially extracted from the plant autumn crocus (Colchicum autumnale), has been used for centuries and is one of the oldest drugs still currently available. The mechanism of action by which colchicine exerts its effects is through the inhibition of tubulin polymerization (14) and potentially also through effects on cellular adhesion molecules and inflammatory chemokines (15). Even when used at low doses, colchicine can be found in white blood cells, blocking tubulin polymerization and subsequent microtubule generation and stability. Through its action on tubulin, colchicine can interfere with many functions of white blood cells including migration and degranulation. Direct inhibition of the migration of neutrophils is considered colchicine's main mechanism of action in gout (16).

Colchicine may also have direct anti-inflammatory effects (17) by inhibiting key inflammatory signaling networks known as the inflammasome and pro-inflammatory cytokines. It has been shown to inhibit membrane expression of adhesion molecules on T cells and endothelial cells (15). Additionally, evidence suggests that colchicine exerts a direct anti-inflammatory effect by inhibiting the synthesis of tumor necrosis factor alpha and IL-6, monocyte migration, and the secretion of matrix metalloproteinase-9 (18, 19). Through the disruption of the cytoskeleton, colchicine is believed to suppress secretion of cytokines and chemokines as well as in vitro platelet aggregation (20, 21).

In the past several years, considerable work has highlighted the potential of colchicine in the treatment of cardiovascular diseases mediated by pro-inflammatory processes. In the COPE (COlchicine for acute PEricarditis) (22), CORE (COlchicine for Recurrent pericarditis) (23) and CORP (COlchicine for Recurrent Pericarditis) (24) trials, colchicine was demonstrated to be effective for the treatment and prevention of recurrent (most likely viral) pericarditis.

Colchicine has also been evaluated for its effect on cardiovascular events in patients with coronary artery disease (CAD). In the LoDoCo Trial, 532 patients with clinically stable CAD were randomly assigned to receive treatment with colchicine (0.5 mg/day) or no colchicine in addition to usual care for a minimum of 2 years (25). Following a mean follow-up of 36 months, colchicine-treated patients experienced significantly fewer cardiovascular events (composite incidence of acute coronary syndrome, out-of-hospital cardiac arrest, or noncardioembolic ischemic stroke) as compared with placebo (5.3% vs. 16.0%; hazard ratio: 0.33; CI 0.18-0.59; p<

0.0001). More recently, we have shown in the COLchicine Cardiovascular Outcomes Trial (COLCOT-1) and published in the New England Journal of Medicine that colchicine at the low dose of 0.5 mg per day significantly reduced the risk of first and total ischemic cardiovascular events by 23% and 34% respectively compared to placebo in 4745 patients with a recent myocardial infarction (26). Through a median follow-up of 23 months in COLCOT-1, diarrhea was reported by 9.7% and 8.9% (P=0.35) and nausea in 1.8% and 1.0% (P=0.02) of patients in the colchicine and placebo groups, respectively. There were more pneumonias (0.9% vs 0.4%, P=0.03) reported as serious adverse events in the colchicine group. This difference in numbers of pneumonias could be due to the play of chance, or could reflect altered immunological responses.

Interestingly, colchicine was shown in one study to reduce hyperoxic lung injury in neonatal rats (27). Indeed, colchicine had favorable effects on alveolarization as well as inflammation and oxidative stress markers in this animal model of broncho-pulmonary dysplasia (27).

Of note, the mechanism of action of colchicine is very different than that of non-steroidal anti-inflammatory drugs. The latter agents inhibit cyclooxygenase and may occasionally shift the use of arachidonic acid towards lipoxygenase, resulting in the production of pro-inflammatory leukotrienes.

1.3 Colchicine Dosage

Pharmascience Inc. was the manufacturer of the 0.5 mg colchicine tablet and matching placebo used in COLCOT-1. Pharmascience Inc. currently manufactures and markets 0.6 mg colchicine tablets in Canada. Tablets of 0.5 mg will be used in the COLCORONA trial.

2 STUDY OBJECTIVES

The primary objective of this study is to determine whether short-term treatment with colchicine reduces the rate of death and lung complications related to COVID-19. The secondary objective is to determine the safety of long-term treatment with colchicine in this patient population. The exploratory objective is to evaluate links between soluble and genetic biomarkers, viral load and treatment effects.

3 STUDY DESIGN

This will be a randomized, double-blind, placebo-controlled study. Following signature of the informed consent form, approximately 6000 subjects meeting all inclusion and no exclusion criteria will be randomized to receive either colchicine or placebo (1:1 allocation ratio) for 30

days. Follow-up phone or video assessments will occur at 15 and 30 days following randomization for evaluation of the occurrence of any trial endpoints or other adverse events.

3.1 Study Schedule

The schedule of visits for this study is outlined in Table 1. However, a patient may be evaluated at any time for safety concerns.

3.2 Enrollment

Informed consent will be obtained from patients who volunteer to participate in the study prior to the conduct of any study-specific procedures. The patient will be considered “enrolled” into the study at the time an informed consent is provided.

Biomarker samples collection will be optional for sites and performed only at sites, selected by the sponsor, where the biomarkers sample collection, storage, and transport to the Beaulieu-Saucier Pharmacogenomics Centre of the Montreal Heart Institute is possible. The informed consent form will reflect this option and patients will specifically be asked to consent to this biomarker evaluation.

Patients who have already signed the informed consent form for the main study will be asked to participate in an optional pharmacogenomic sub-study. If they agree, they will be asked to sign the pharmacogenomic informed consent form and a signed copy will be provided to them. Frozen blood samples will be kept for future analysis of responses to colchicine and genes involved in respiratory, cardiovascular, and related diseases.

3.3 Screening Evaluations and Randomization

Screening evaluations will include a review of the patient’s medical history and assessment of concomitant medications to determine if the patient qualifies for the study. Patients who meet all inclusion criteria and no exclusion criteria will be randomized to receive the study drug or placebo. All patients must be randomized within 2 days of the diagnosis of COVID-19 infection.

There is no wash-out period required for patients who have been treated with colchicine and stopped treatment prior to enrolment.

Women of childbearing potential must have a negative urine pregnancy test result at the time of randomization in order to qualify. Qualifying patients will be randomized to receive placebo or colchicine administered in a blinded manner. Blinded randomization will be performed through an automated Interactive Web Response System (IWRS). 3000 patients will be randomized to active treatment and 3000 patients will be randomized to placebo for a total of 6000 randomized patients.

3.4 Active Treatment Period

All patients will receive study medication (either colchicine 0.5 mg or matching placebo) per os (PO) twice daily for the first 3 days and then once daily for the last 27 days. If a dose is missed, it should not be replaced. Throughout the study, patients will undergo two videovisits and/or phone contacts (at 15 and 30 days) to assess for potential study endpoints and other adverse events (AEs). Patients will be dispensed study medication for the entire follow-up period of 30 days. At each videovisit and/or phone contact, patients will be 1) questioned in a non-specific manner for the occurrence of AEs and any change in concomitant medications; and 2) encouraged to comply with the study protocol including adherence to study medication.

Review of routine chemistry and hematology parameters will be done at Visit 2 where available in the patient's record.

Blood draws for biomarker samples are optional for sites, depending on their ability to collect, store, and transport samples to the Beaulieu-Saucier Pharmacogenomics Centre of the Montreal Heart Institute. At these sites only, patients who agree to and have signed the biomarkers informed consent form will participate in the optional biomarkers sub-study.

At Visit 2, an optional blood draw for the pharmacogenomic sub-study may be performed at sites that have the ability to collect, store and transport samples to the Beaulieu-Saucier Pharmacogenomics Centre of the Montreal Heart Institute. At these sites only, patients who agree to and have signed the pharmacogenomic informed consent form will participate in the optional pharmacogenomic sub-study.

Please refer to Table 1 and Appendix A for Timetable of Visits and Procedures.

Table 1 Timetable of Visits and Procedures
(Note: all visits are to be +/- 2 days from anticipated timepoint)

**Visits**	**1 Screening Visit (on-site)**	**2[a] Randomization/ Baseline**	**3[b] Phone or video contact**	**4[b] Phone or video End of Study**
**Days**		**0**	**15**	**30**
Informed consent	X			
Medical/Surgical history	X			
Phone contact			X	X
Review Inclusion/Exclusion criteria	X	X		
Urine pregnancy test (only women of childbearing potential)		X		
Randomization		X		
Blood draw for biomarkers (optional)		X		
Blood draw for pharmacogenomics evaluation (optional)		X [c]		
Record potential study endpoints and other AEs		X	X	X
Study medication dispensing		X		

a) Screening Visit and Visit 2 may be performed on the same day; under these circumstances, procedures to be performed at both Screening Visit and Visit 2 will be performed once.
b) Phone contact or video visit.
c) Blood draw for pharmacogenomics evaluation can be collected at the next in-person visit if randomization visit has already occurred at the time of the implementation of the sub-study at the site.

4 STUDY POPULATION

4.1 Source and Number of Patients

A total of 6000 patients will be randomized.

4.2 Patient Selection Criteria

4.2.1 Inclusion Criteria:

All of these criteria must be met:

- Males and females, at least 40 years of age, capable and willing to provide informed consent;
- Patient must have received a diagnosis of COVID-19 infection within the last 24 hours;
- Outpatient setting (not currently hospitalized or under immediate consideration for hospitalization);
- Patient must possess at least one of the following high-risk criteria: 70 years or more of age, diabetes mellitus, uncontrolled hypertension (systolic blood pressure ≥150 mm Hg), known respiratory disease (including asthma or chronic obstructive pulmonary disease), known heart failure, known coronary disease, fever of ≥38.4°C within the last 48 hours, dyspnea at the time of presentation, bicytopenia, pancytopenia, or the combination of high neutrophil count and low lymphocyte count;
- Female patient is either not of childbearing potential, defined as postmenopausal for at least 1 year or surgically sterile, or is of childbearing potential and practicing at least one method of contraception and preferably two complementary forms of contraception including a barrier method (e.g. male or female condoms, spermicides, sponges, foams, jellies, diaphragm, intrauterine device (IUD)) throughout the study and for 30 days after study completion;
- Patient must be able and willing to comply with the requirements of this study protocol.

4.2.2 Exclusion Criteria:

None of these exclusion criteria should be met:

- Patient currently hospitalized or under immediate consideration for hospitalization;
- Patient currently in shock or with hemodynamic instability;
- Patient with inflammatory bowel disease (Crohn’s disease or ulcerative colitis) or patient with chronic diarrhea;
- Patient with pre-existent progressive neuromuscular disease;
- Estimate Glomerular filtration rate (eGFR) using the MDRD equation for all subjects being considered for enrollment, with a cut-off of < 30 mL/m in/1.73m^2.

- Patient with a history of cirrhosis, chronic active hepatitis or severe hepatic disease;
- Female patient who is pregnant, or breast-feeding or is considering becoming pregnant during the study or for 6 months after the last dose of study medication;
- Patient currently taking colchicine for other indications (mainly chronic indications represented by Familial Mediterranean Fever or gout). There is no wash-out period required for patients who have been treated with colchicine and stopped treatment prior to enrolment;
- Patient with a history of an allergic reaction or significant sensitivity to colchicine;
- Patient undergoing chemotherapy for cancer;
- Patient is considered by the investigator, for any reason, to be an unsuitable candidate for the study.

4.3 Prohibited, Allowable and Concurrent Medications

Colchicine is a substrate of intestinal and hepatic cytochrome P450 3A4 (CYP3A4), which catalyzes demethylation of colchicine to inactive metabolites. Consistent with the current understanding of colchicine metabolism, certain drugs increase the potential for colchicine toxicity via modulation of the efflux transporter P-glycoprotein (P-gp) and CYP3A4 activity. As such, during the duration of the trial, the concomitant use of erythromycin,clarithromycin, cyclosporine and verapamil is prohibited. Toxicity has also been reported in a patient who began to regularly consume a liter of grapefruit juice daily while being treated chronically with colchicine, and therefore the concomitant consumption of grapefruit juice () is to be avoided during the study.

It is recommended that subjects who are taking other moderate or strong inhibitors of CYP3A4, or drugs that compete for the P-gp transporter should be considered for enrollment on a case by case basis based on the potential for the accumulation of colchicine and resultant toxicity.

All other medications (including oral or parenteral hormonal contraceptive agents) are allowed as long as they are stabilized prior to study entry and maintained as stable throughout the course of the trial.

The use of concomitant medications at the time of randomization will be recorded in the eCRF (with the exceptions of concomitant medication taken “when necessary” (PRN)).

5 STUDY METHODOLOGY

5.1 Efficacy Outcomes

The primary endpoint will be the composite of death or the need for hospitalization due to COVID-19 infection in the first 30 days after randomization. The secondary endpoints will

consist of the components of the composite primary endpoint; and the need for mechanical ventilation in the first 30 days after randomization. Exploratory endpoints will include associations between both biomarkers and viral load (stored samples) and treatment effects.

Biomarkers endpoints:
Samples received from participating sites will be kept frozen for future use for evaluation of biomarkers and the response to treatment, possibly including but not limited to markers of inflammation.

Additional exploratory endpoints will include these biomarkers at Visit 2. Frozen samples will be kept at the Beaulieu-Saucier Pharmacogenomics Centre of the Montreal Heart Institute.

5.2 Safety Monitoring

Drug safety will be assessed by an evaluation of types, frequencies, severities and duration of any reported AEs. Patients will be monitored for signs and symptoms of drug toxicity.

Appendix B includes the product monograph of pms-colchicine 0.6 mg tablets, the product currently marketed by Pharmascience Inc. in Canada for gout therapy. Investigators should consult this information and their locally approved prescribing information (if available) to seek guidance regarding potential colchicine toxicity and its potential signs and symptoms.

For all toxicities that require the study therapy to be temporarily or permanently discontinued, relevant clinical and laboratory tests will be repeated as clinically needed until there is final resolution, stabilization of the toxicity, or another cause for the abnormality is determined.

5.2.1 Adverse Event Reporting

Information regarding AEs will be collected from the first dose of study medication (at Visit 2) through and including the last visit. Any AEs prior to randomization will be recorded in the medical history and kept in the patients' chart.

In addition to standard reporting of Serious Adverse Events (SAEs) as defined and outlined in Section 5.2.1.1 below, information regarding SAEs that occur within 30 days following the last study visit, and reported to the investigational site, will be collected. Each patient will be observed and queried in a non-specific fashion at each visit during the study for any new or continuing symptoms since the previous visit.

All SAEs will be recorded on the appropriate eCRF section. The only other AEs to be recorded on the eCRF are those that are either related to the gastrointestinal system, that are judged related to the study medication by the investigator or laboratory abnormalities judged clinically significant by the investigator. Information collected will include the onset, duration, severity, relationship to study drug, and the management as outlined in Appendix C.

5.2.1.1 Serious Adverse Events

Serious Adverse Events (SAE) are those that meet any of the following International Council for Harmonisation (ICH) criteria:

- Is fatal or immediately life-threatening;
- Results in persistent or significant disability/incapacity;
- Requires or prolongs inpatient hospitalization;
- Is a congenital anomaly/birth defect in the offspring of the patient;
- Is a cancer;
- Is an overdose (intentional or accidental);
- Is judged to be medically important.

Medically important events may not be immediately life-threatening or result in death or hospitalization but may jeopardize the patient or may require intervention to prevent one of the outcomes listed in the definition above. Serious Adverse Events are to be reported if they are known to occur within 30 days following the last study visit.

Medical and scientific judgment should be exercised in deciding whether other AEs meet these criteria and are immediately reportable to the sponsor or designee.

In the event of a serious or life-threatening adverse event, or in the event of death, immediately report the event on the appropriate SAE form in the eCRF.

FOR ANY SAFETY QUESTIONS OR CONCERNS, PLEASE CONTACT:
MHICC Medical Monitor
Phone: +1-514 207-1365 (24/7 availability)
Email: colcorona_safety@mhicc.org

If any SAE occurs, the study treatment may be interrupted or discontinued at the Investigator's discretion. If an acute medical emergency occurs, the investigator should make every effort to reach the contact person listed above, before breaking the randomization code, via the automated Interactive Web Response System (IWRS). However, the investigator may break the randomization code (via IWRS) at any time if this is required for proper treatment of the patient and even if the contact person could not be reached.

The MHICC Medical Monitor or his designated representative is responsible to report any Suspected Unexpected Serious Adverse Reaction (SUSAR) to the regulatory authorities and to copy Pharmascience Inc. at time of submission. Reports to the Health Authorities must be made within 7 calendar days, (for death and life-threatening events) and within 15 calendar days (for other serious events) after being informed of an SAE by the investigator.

5.2.1.2 Lack of Efficacy of study medication

Any signs or symptoms defined as lack of efficacy or collected as efficacy parameters (i.e.: study endpoints including hospitalizations) will not be reported as SAEs, but must be included as an Adverse Event in the database.

5.2.1.3 Adverse Event Follow-Up

Record all reportable serious and non-serious AEs on the appropriate eCRF including the onset, duration, severity, relationship to the study drug, and ultimate management. All AEs reported during the treatment phase should be recorded and followed until the AE has subsided, or stabilized or until the end of the study, whichever occurs first. The study will be stopped at any time if new knowledge is gained and the risk-benefit ratio is no longer favorable for the participating patients and Pharmascience Inc. will then be informed immediately but no later than 3 business days if such decision is made.

5.2.2 Other Safety Monitoring

5.2.2.1 Laboratory Evaluations

Chemistry and hematology test results (refer to Appendix A) obtained for a clinical indication may be used to assess patient eligibility at screening.

In participating sites, optional biomarker blood samples and respiratory samples for viral load will be collected at Visit 2 and kept at the Montreal Heart Institute for up to five (5) years following completion of the trial for possible future evaluation of biomarkers. These samples may be used to assess (but not limited to) markers of inflammation and markers of oxidative stress.

In participating sites, optional blood samples for the pharmacogenomic sub-study will be collected at Visit 2 from patients who have signed the pharmacogenomic informed consent form. Any patient already enrolled in the trial at the time of implementation of the sub-study will be offered to participate in the pharmacogenomic sub-study at any on-site visit. Blood samples for pharmacogenomic evaluation may be kept at the Beaulieu-Saucier Pharmacogenomics Centre of the Montreal Heart Institute for up to 20 years following completion of the trial to conduct future analysis of responses to colchicine and genes involved in cardiovascular, lung and related diseases. If, upon reception of the pharmacogenomic samples at the Beaulieu-Saucier Pharmacogenomics Centre of the Montreal Heart Institute, the sample cannot be analyzed because of DNA degradation, a second blood draw will be requested.

5.3 Withdrawal of Patients from the Study

Patients have the right to withdraw from the study at any time during the course of the study. However, every effort should be made, within the bounds of safety, patient choice and the provisions of informed consent, to have each patient complete the study up to and including the last protocol-specified study visit. If the study medication jeopardizes the patient’s health or if the patient wishes to discontinue for any reason, study medication can be discontinued but the

patient should be encouraged to remain in the study for the follow-up visits up to the End of Study visit. If the patient does not wish to pursue the protocol follow-up visits, the option for a last contact at the end of the study to obtain their vital status should be proposed. Patients who are not compliant during the active treatment period should be counseled on the importance of complying with study requirements and be allowed to remain in the study. No patient who has withdrawn their consent from the study during the active treatment period should be replaced.

In the case of an adverse event or safety concern, the study medication may be withheld temporarily, or the dose reduced, as per investigator judgment. Investigators should consult the pms-colchicine product monograph (Appendix B) and discuss the patient condition with the MHICC medical monitor in order to determine the appropriate dose reduction algorithm or study medication discontinuation plan.

Patients withdrawn at any time from the study during the active treatment period should complete all last protocol-specified visit procedures (End of Study Visit, the reason for withdrawal from the study, the date of the last visit and the date of the last dose of double-blind medication will be clearly documented in the eCRF).

5.4 Study Completion

The study will end when the last randomized patient will have completed his (or her) 30 day follow-up phone contact or videovisit. Completion of the study by a patient should be clearly indicated in the eCRF, along with the date of the last visit and the date of the last dose of double-blind medication.

6 STUDY MEDICATION

Pharmascience Inc. will provide both colchicine and placebo in bottles of 34 tablets of 0.5 mg each. At the time of randomization, the randomization number will be recorded on the appropriate eCRF section. Blinded medication will be provided as 0.5 mg colchicine tablets or matching placebo tablets. A detailed set of dispensing instructions will be included with the drug shipment.

6.1 Medication Dispensing

Study medication may be dispensed by the designated pharmacist or a qualified investigative site representative, according to a detailed set of dispensing instructions. Study medication may also be shipped directly to the patient’s home depending on evolving circumstances related to COVID-19.

6.2 Dosage Regimen

At randomization, patients will be dispensed colchicine tablets or placebo to match colchicine tablets. Patients will be instructed to take tablets twice daily for the first 3 days and then once a

day for the last 27 days and according to the detailed set of dispensing instructions outlined on the label.

7 DATA COLLECTION

Electronic Case Report Forms (eCRF) for all patients will be supplied by MHICC. These are to be completed as instructed. Original source documents and other study documentation will be maintained at the study site. Risk-based monitoring will be performed by the MHICC or its designated representative.

8 DATA ANALYSIS AND STATISTICAL CONSIDERATIONS

8.1 Statistical Power and Sample Size Considerations

All main analyses in COLCORONA will be conducted on an intention-to-treat basis. A sample size of approximately 6000 randomized patients with 3000 patients in each treatment group will be required to detect a 25% risk reduction with colchicine with a power of 80% given a primary endpoint event rate of 7% in the placebo group and a two-sided test at the 0.05 significance level.

The final analysis of the primary endpoint will be conducted at a significance level slightly below the 0.05 level to account for the interim analysis (see below). However, since this will have a negligible impact on power, the sample size calculation was calculated using a significance level of 0.05.

8.2 Analysis population

8.2.1 Intent to Treat (ITT) population

All patients randomized will be included in the ITT population. Patients will be assigned to treatment groups as randomized for analysis purposes.

8.2.2 Safety population

All patients who received at least one dose of study medication will be included in the safety analysis population. Patients will be assigned according to the true treatment received for analysis purposes.

8.3 Data analysis

8.3.1 Analysis of efficacy outcomes

The primary analysis of efficacy will be based on the intent-to-treat principle. The primary endpoint will be compared between the two treatment groups using a chi-square test. Logistic regression models might also be used to compare the primary endpoint between the two

treatment groups while accounting for important baseline characteristics. Secondary endpoints will be analyzed similarly. Exploratory analysis (biomarkers) will include logistic regression models with the primary/secondary endpoints as dependent variables and with treatment group, biomarker and treatment group x biomarker interaction as independent variables.

All statistical tests will be two-sided and conducted at the 0.05 significance level, with the exception of the primary analysis that will be conducted at a slightly lower level to account for the interim analysis. Statistical analyses will be done using SAS version 9.4 or higher.

8.3.2 Analysis of safety outcomes

Safety of colchicine will be evaluated by presenting descriptive statistics for various safety endpoints broken down by group. This will be done for the population of patients who received at least one dose of study medication (safety population).

8.3.3 Interim analysis

A fully independent 5-member Data and Safety Monitoring Board (DSMB) will be established and will review unblinded safety data as detailed in the DSMB charter. An interim analysis is planned after approximately 50% of randomized patients have completed 30 days of follow-up. The DSMB charter will pre-specify the methods of interim efficacy analyses and the rules for early study termination, approved by all board members. The stopping rules for efficacy and futility will be based on the O'Brien-Fleming alpha-spending function or on a similar conservative approach so that the impact on the final alpha level will be negligible. The DSMB will have the option of recommending early study termination because of overwhelming efficacy, early termination for futility, or continuation of the trial as planned.

The final analysis of the primary endpoint will be conducted at a significance level slightly below the 0.05 level to account for the interim analysis. However, since this will have a negligible impact on power, the sample size calculation was calculated using a significance level of 0.05.

9 STUDY COORDINATION

The MHICC will be responsible for processing and quality control of the data. Project management will be carried out as described in the MHICC standard operating procedures (SOPs) for clinical studies. The handling of data, including data quality control, will comply with all applicable regulatory guidelines, MHICC SOPs and the study Data Management Plan.

REFERENCES

1. Zhu N, Zhang D, Wang W, Li X, Yang B, Song J, et al. A novel coronavirus from patients with pneumonia in China, 2019. N Engl J Med 2020.doi.org/10.1056/ NEJMoa2001017

2. Huang C, Wang Y, Li X, Ren L, Zhao J, Hu Y, et al. Clinical features of patients infected with 2019 novel coronavirus in Wuhan, China. Lancet 2020 doi.org/10.1016/S0140-6736(20)30183-5.

3. Wang D, Hu B, Hu C, Zhu F, Liu X, Zhang J, et al. Clinical characteristics of 138 hospitalized patients with 2019 novel coronavirus-infected pneumonia in Wuhan, China. JAMA 2020 doi.org/ 10.1001/jama.2020.1585.

4. Tian S, Hu W, Niu L, Liu H, Xu H, Xiao S-Y. Pulmonary pathology of early phase 2019 novel coronavirus (COVID-19) pneumonia in two patients with lung cancer, J Thor Oncology 2020 doi: https://doi.org/10.1016/j.jtho.2020.02.010.

5. Hoffmann M, Kleine-Weber H, Schroeder S, et al. SARS-CoV-2 cell entry depends on ACE2 and TMPRSS2 and is blocked by a clinically proven protease inhibitor. Cell 2020;181:1-10.

6. Zhang H, Baker A. Recombinant human ACE2: acing out angiotensin II in ARDS therapy. Critical Care 2017;21:305-307.

7. Mehta P, McAuley DF, Brown M, Emilie Sanchez E, Tattersall RS, Jessica J Manson JJ. COVID-19: consider cytokine storm syndromes and immunosuppression. Lancet 2020 March 13 https://doi.org/10.1016/ S0140-6736(20)30628-0.

8. Fedson DS. Treating the host response: an alternative way to manage Ebola in Africa and the next influenza pandemic. JOGH 2019;9: doi: 10.7189/jogh.09.010322.

9. Fedson DS. Influenza, evolution, and the next pandemic. Evolution, Medicine, and Public Health 2018;260-269.

10. Barsness KA, Bensard DD, Partrick DA, Calkins CM, Hendrickson RJ, McIntyre RC Jr. Endotoxin induces an exaggerated interleukin-10 response in peritoneal macrophages of children compared with adults. J Pediatr Surg 2004;39:912-5.

11. Barsness KA, Bensard DD, Partrick DA, et al. IL-1beta induces an exaggerated pro- and anti-inflammatory response in peritoneal macrophages of children compared with adults. Pediatr Surg Int 2004;20:238-42.

12. Hwang W, Lee CG, Lee C, et al. Locus-specific reversible DNA methylation regulates transient IL-10 expression in Th1 cells. J Immunol 2018;200:1865-75.

13. Ip WKE, Hoshi N, Shouval DS, Snapper S, Medzhitov R. Anti-inflammatory effect of IL-10 mediated by metabolic reprogramming of macrophages. Science 2017;356:513-19.

14. Ravelli RBG, Gigant B, Curmi PA, et al. Insight into tubulin regulation from a complex with colchicine and a stathmin-like domain. Nature 2004;428:198-202.

15. Perico N. Ostermann D, Bontempeill M, et al. colchicine interferes with L-selectin and leukocyte function associated antigen-1 expression on human T lymphocytes and inhibits T cell activation. J Am Soc Nephrol 1996;7:594-601.

16. Matsukawa A, Yoshimura T, Maeda T, et al. Analysis of the cytokine network among tumor necrosis factor alpha, interleukin-1beta, interleukin-8 and interleukin-1 receptor antagonist in monosodium urate crystal-induced rabbit arthritis. Lab Investig J Techn Methods Pathol 1998; 78:559-569.

17. Pope RM, Tschopp J. The role of interleukin-1 and the inflammasome in gout: implications for therapy. Arthritis Rheumat 2007;56(10):3183-3188.

18. Cerquaglia C, Diaco M, Nucera G, et al. Pharmacological and clinical basis of treatment of familial Mediterranean fever (FMF) with colchicine or analogues: an update. Curr Drug Targ Inflamm Allergy 2005;4:117-124.

19. Wesley II RB, Meng X, Godin D, Galis ZS. Extracellular matrix modulates macrophage functions characteristic to atheroma: collagen type I enhances acquisition of resident macrophage traits by human peripheral blood monocytes in vitro. Arterioscler Thromb Vasc Biol 1998;18: 432-440.

20. Chao FC, Shepro D, Tullis JL, Belamarich FA, Curby WA. Similarities between platelet contraction and cellular motility during mitosis: role of platelet microtubules in clot retraction. J Cell Sci 1976;20:569-588.

21. Sneddon JM. Effect of mitosis inhibitors on blood platelet microtubules and aggregation. J Physiol 1971;214:145-158.

22. Imazio M, Bobbio M, Cecchi E, et al. Colchicine in addition to conventional therapy for acute pericarditis: results of the Colchicine for acute Pericarditis (COPE) trial. Circulation 2005; 112:2012-2016.

23. Imazio M, Bobbio M, Cecchi E, et al. Colchicine as first-choice therapy for recurrent pericarditis: results of the CORE (Colchicine for Recurrent pericarditis) trial. Arch Intern Med 2005;165:1987-1991.

24. Imazio M, Brucato A, Cemin R, et al. Colchicine for recurrent pericarditis (CORP): a randomized trial. Ann Intern Med 2011;155:409-414.

25. Nidorf SM, Eikelboom JW, Budgeon CA, Thompson PL. Low-dose colchicine for secondary prevention of cardiovascular disease. J Am Coll Cardiol 2013;61:404-410.

26. Tardif JC, Kouz S, Waters DD, et al. Efficacy and safety of low-dose colchicine after myocardial infarction. N Engl J Med 2019;381:2497-2505.

27. Ozdemir R, Yurttutan S, Talim B, Uysal B, Erdeve O, Oguz SS, Dilmen U. Colchicine protects against hyperoxic lung injury in neonatal rats. Neonatology 2012;102:265–269

LIST OF APPENDICES

**Appendix**	**Title**
A	Detailed Study Safety Parameters
B	PMS-Colchicine 0.6mg Product Monograph
C	Administrative Procedures for the Reporting of Adverse Events
D	Other Administrative and Regulatory Procedures
E	Declaration of Helsinki

Appendix A Detailed Study Safety Parameters

1. Medical/Surgical History

The following elements of medical/surgical history will be recorded at the Screening Visit:

- age
- ethnic origin
- sex
- smoking history
- history of diabetes
- history of hypertension
- history of dyslipidemia
- prior MI
- prior PCI
- prior CABG
- prior stroke
- prior heart failure
- prior obstructive pulmonary disease
- prior other respiratory disease

2. Physical Appearance

1. weight
2. height
3. waist circumference

3. Laboratory Parameters to Assess at V1 or V2

Chemistry	AST (Aspartate aminotransferase)<br>ALT (Alanine transaminase)<br>GGT (Gamma-glutamyl transferase)<br>Alkaline phosphatase<br>Total Bilirubin<br>Serum creatinine<br>Uric acid

Appendix B **PMS-Colchicine 0.6mg Product Monograph**

PRESCRIBING INFORMATION

[Pr] pms-COLCHICINE

Colchicine Tablets, USP

0.6 mg

Gout Therapy

PHARMASCIENCE INC.
6111 Royalmount Ave., Suite 100
Montreal, Québec
H4P 2T4

www.pharmascience.com

Date of Revision:
July 05, 2016

Control No.: **196333**

Pr pms-COLCHICINE 0.6 mg per Tablet
Gout Therapy
Preparations with No Effect on Uric Acid Metabolism

Structural Formula:

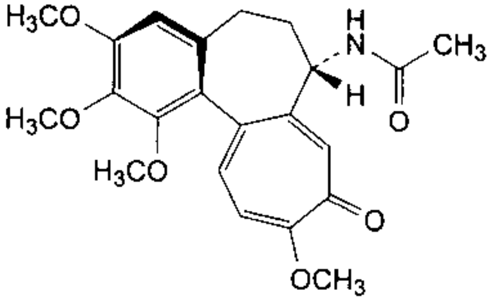

Molecular formula: $C_{22}H_{25}NO_6$

Molecular Mass: 399.4 g/mol

PHARMACOLOGY

Colchicine is an alkaloid extracted from plants of the genus Colchicum (*Colchicum automnale*) and is a water soluble pale yellow powder which blackens with exposure to light.

Oral colchicine intake undergoes an entero-hepatic cycle. It is absorbed rapidly by the Gastro-Intestinal Tract. The drug and its metabolites are distributed in the leukocytes, the kidneys, the liver, the spleen and the intestine.

Peak plasma concentration is obtained from 0.5 to 2 hours after ingestion. The half-life of this drug is approximately 20 minutes in the plasma and 60 hours in the leucocytes. The drug is 50% bound to proteins. The interleucocyte concentrations are higher than the concentration in the plasma.

Its metabolism is not well understood. Colchicine is metabolized in the liver and is excreted mainly in the feces, 10-20% of the drug finds its way in the urine. The colchicine binding results in its accumulation in tissues as soon as the daily dosage exceeds 1 mg, which, in turn, could result in toxic effects. A serious renal ailment could prolong the half-life for its elimination.

Colchicine crosses the placenta and passes into the breast milk.

Although its exact mode of action in the relief of gout is not completely understood, colchicine is known to decrease the inflammatory response to urate crystal deposition by inhibiting migration of leukocytes, to interfere with urate deposition by decreasing lactic acid production by leukocytes, to interfere with kinin formation and to diminish phagocytosis and the subsequent anti-inflammatory response.

The anti-inflammatory effect of colchicine is relatively selective for acute gouty arthritis. It is neither analgesic nor a uricosuric and will not prevent progression to chronic gouty arthritis. It does have a prophylactic, suppressive effect that helps to reduce the incidence of acute attacks. .

Comparative bioavailability study

A pivotal, single center, randomized, single dose, double-blinded, two-sequence, two-treatment, crossover design study was conducted in 34 healthy male volunteers under fasting conditions comparing pms-COLCHICINE 0.6 mg tablets (Pharmascience Inc.) to COLCHICINE-ODAN 0.6 mg tablets (Odan Laboratories Ltd.). A summary of the pharmacokinetic data is presented in the following tables.

SUMMARY TABLE OF THE COMPARATIVE BIOAVAILABILITY DATA

Colchicine<br>(1 x 0.6 mg tablet – Fasting Conditions)<br>From measured data **uncorrected for potency** Geometric Mean<br>Arithmetic Mean (CV %)				
Parameter	Test*	Reference†	% Ratio of Geometric Means	90 % Confidence Interval
AUC_{0-72} (pg·h/mL)	15457.6<br>16029.3 (25.8)	16324.5<br>16713.6 (21.8)	94.69	89.53 – 100.14
AUC_I (pg·h/mL)	17827.6<br>18487.9 (26.2)	18702.9<br>19154.7 (22.1)	95.32	90.25 – 100.67
C_{max} (pg/mL)	2068.1<br>2208.7 (35.4)	2130.8<br>2216.6 (27.9)	97.06	88.39 – 106.57
T_{max}§ (h)	1.05<br>(0.50 – 2.50)	1.00<br>(0.75 – 2.03)		
$T_{½}$€ (h)	29.91 (17.5)	29.68 (15.8)		

* pms-COLCHICINE 0.6 mg tablets, Pharmascience Inc., Montreal, Quebec, Canada
† Colchicine 0.6 mg tablets, Odan Laboratories Ltd., Montreal, Quebec, Canada
§ Median (range)
€ Arithmetic mean (CV%)

INDICATIONS

- Gout: prophylaxis and treatment of gout flares in adults. Colchicine is effective in relieving the pain of acute attacks, especially if therapy is begun early in the attack and in adequate dosage. Many therapists use colchicine as interval therapy to prevent acute attacks of gout.
- Familial Mediterranean Fever (familial recurrent polyserositis).

CONTRAINDICATIONS

- Patients with serious gastrointestinal, hepatic, renal and cardiac disease.
- Patients with known hypersensitivity to colchicine.
- Patients with renal or hepatic impairment should not be given pms-COLCHICINE in conjunction with P-glycoprotein or strong CYP3A4 inhibitors.
- Avoid pms-COLCHICINE plus cyclosporine or a strong 3A4 inhibitor if patients have renal or hepatic impairment. This can be fatal.

WARNINGS AND PRECAUTIONS

Warnings:

Drug Interactions

Colchicine is a substrate for both the cytochrome P450 3A isoform subfamily (CYP3A) and the efflux transporter, P-glycoprotein (P-gp). Clarithromycin and other macrolides are known to inhibit CYP3A4 and P-gp. When colchicine and clarithromycin are administered together, inhibition of P-gp and/or CYP3A4 by clarithromycin may lead to increased exposure to colchicine which could result in clinically significant safety concerns. Patients should be monitored for clinical symptoms of colchicine toxicity. There have been post-marketing reports of colchicine toxicity with concurrent use of colchicine and clarithromycin.

In patients with impaired renal function and/or who are elderly, colchicine and clarithromycin should not be used concurrently due to the risk of colchicine toxicity. Deaths have been reported in some of these patients.

Colchicine has been shown to induce reversible malabsorption of Vitamin B_{12}, apparently by altering the function of ileal mucosa.

Blood dyscrasias: myelosuppression, leucopenia, ganulocytopenia, thrombocytopenia, and aplastic anemia have been reported with colchicine used in therapeutic doses.

Pregnancy: Cell division in animals and plants can be arrested by colchicine. In certain species of animal under certain conditions it has produced teratogenic effects and has adversely affected spermatogenesis. If the drug is used during pregnancy, or if the patient becomes pregnant while taking it, the woman should be told of the potential hazard to the fetus. While such effects have not been demonstrated in humans, pertinent available information is meager. In view of the above, colchicine is not recommended for use in pregnancy.

Neuromuscular Toxicity: Colchicine-induced neuromuscular toxicity and rhabdomyolysis have been reported with chronic treatment in therapeutic doses. Patients with renal dysfunction and elderly patients, even those with normal renal and hepatic function, are at increased risk. Concomitant use of atorvastatin, simvastatin, pravastatin, fluvastatin, gemfibrozil, fenofibrate, fenofibric acid, or benzafibrate (themselves associated with myotoxicity) or cyclosporine with colchicine may potentiate the development of myopathy. Once colchicine is stopped, the symptoms generally resolve within 1 week to several months.

Precautions:

Periodic blood tests are suggested since prolonged administration of colchicine could cause blood dyscrasias.

Colchicine is a toxic substance and must be given only under physician's care. Since the administration of colchicine is subjected to wide variations, the prescribed dosage must be strictly followed.

Use with care in geriatrics or debilitated patients and those with cardiac, renal or gastrointestinal disease. Dosage reduction may be necessary in these cases and is indicated if weakness, anorexia, nausea, vomiting or diarrhea appears (see Contraindications).

Pregnancy: pms-COLCHICINE is not recommended for use in pregnancy (see Warnings).

Lactation: It is not known if colchicine is distributed into human milk. pms-COLCHICINE should not be used by women who are breastfeeding.

Children: Safety and efficacy of colchicine in treatment of FMF have not been established in patients ≤12 years of age. Colchicine is not recommended in pediatric patients for treatment or prophylaxis of gout flares. Keep colchicine out of reach of children. Fatal overdoses, both accidental and intentional, have been reported in adults and children who have ingested colchicine.

ADVERSE EFFECTS

Reactions to colchicine appear to be dose related. The most prominent symptoms are referable to the gastrointestinal tract (e.g. nausea, vomiting, abdominal pain, diarrhea) and may be particularity troublesome in the presence of peptic ulcer or spastic colon. At toxic doses colchicine may cause severe diarrhea, generalized vascular damage, and renal damage, with hematuria and oliguria. Muscular weakness, which disappears with discontinuance of therapy, urticaria, dermatitis, and purpura have also been reported. Hypersensitivity to colchicine is a very rare occurrence, but it should be borne in mind. The appearance of any of the aforementioned symptoms may require reduction of dosage or discontinuance of the drug. When given for a prolonged period, colchicine may cause agranulocytosis, aplastic anemia, peripheral neuritis and loss of hair.

There have been post-marketing reports of colchicine toxicity with concomitant use of clarithromycin and colchicine, especially in the elderly, some of which occurred in patients with renal insufficiency. Deaths have been reported in some such patients (see Warnings).

Post-marketing Experience

Serious toxic manifestations associated with colchicine include myelosuppression, disseminated intravascular coagulation, and impairment of renal, hepatic, circulatory, and central nervous systems.

The following adverse reactions have been reported with colchicine. These have been generally reversible upon temporarily interrupting treatment or lowering the dose of colchicine.

Neurological: sensory motor neuropathy

Dermatological: alopecia, maculopapular rash, purpura, rash

Digestive: abdominal cramping, abdominal pain, diarrhea, lactose intolerance, nausea, vomiting

Hematological: leukopenia, granulocytopenia, thrombocytopenia, pancytopenia, aplastic anemia

Hepatobiliary: elevated AST, elevated ALT

Musculoskeletal: myopathy, elevated CPK, myotonia, muscle weakness, muscle pain, rhabdomyolysis

Reproductive: azoospermia, oligospermia

Reporting Side Effects
You can help improve the safe use of health products for Canadians by reporting serious and unexpected side effects to Health Canada. Your report may help to identify new side effects and change the product safety information.
3 ways to report:

- Online at MedEffect (http://www.hc-sc.gc.ca/dhp-mps/medeff/index-eng.php);;
- By calling 1-866-234-2345 (toll-free);
- By completing a Consumer Side Effect Reporting Form and sending it by:
  - Fax to 1-866-678-6789 (toll-free), or
  - Mail to: Canada Vigilance Program
    Health Canada, Postal Locator 0701E
    Ottawa, ON
    K1A 0K9

  Postage paid labels and the Consumer Side Effect Reporting Form are available at MedEffect (http://www.hc-sc.gc.ca/dhp-mps/medeff/index-eng.php).

NOTE: Contact your health professional if you need information about how to manage your side effects. The Canada Vigilance Program does not provide medical advice.

DOSAGE AND ADMINISTRATION

Dosage

Gout Flares:

Treatment of gout flares in adults (>18 years old):

The recommended dose of pms-COLCHICINE is 1.2 mg (2 tablets) at the first sign of the flare followed by 0.6mg (1 tablet) one hour later.

The maximum recommended dose is 1.8 mg over one-hour period. Wait 12 hours to resume prophylactic dose. Wait at least three days to repeat.

The treatment of gout flares with pms-COLCHICINE is not recommended in patients receiving prophylactic dose of colchicine and CYP3A4 inhibitors.

Prophylaxis of gout flares in adults (>18 years old):

The recommended dosage of pms-COLCHICINE is 0.6 mg once or twice daily. The maximum recommended dose should not exceed 1.2 mg per day. Use with caution in geriatric patients; reduce prophylactic daily dose by 50% in individuals >70 years.

Familial Mediterranean Fever (FMF):

Treatment of FMF in patients >12 years of age:

The recommended dosage of colchicine for FMF is 1.2 mg to 2.4 mg daily.

The dosage should be increased as needed to control disease and as tolerated in increments of 0.3 mg/day to a maximum recommended daily dose of 2.4 mg. If intolerable side effects develop, the dose should be decreased in increments of 0.3 mg/day. The total daily dose of pms-COLCHICINE may be administered in one to two divided doses.

Dose Modification for Co-administration of Interacting Drugs

Concomitant Therapy:

Co-administration of colchicine with drugs known to inhibit CYP3A4 and/or P-glycoprotein (P-gp) increases the risk of colchicine-induced toxic effects (Table 1). If patients are taking or have recently completed treatment with drugs listed in Table 1 within the prior 14 days, the dose adjustments are as shown on the table below (see Warnings and Drug Interactions).

Table 1: Colchicine Dose Adjustment for Co-administration with Interacting Drugs if no Alternative Available[1]

Drug	Noted or Anticipated Outcome	Gout Flares				FMF	
		Prophylaxis of Gout Flares		Treatment of Gout Flares			
		Original Intended Dosage	Adjusted Dose	Original Intended Dosage	Adjusted Dose	Original Intended Dosage	Adjusted Dose
**Strong CYP3A4 Inhibitors[2]**							
Atazanavir<br>Clarithromycin<br>Darunavir/ Ritonavir[3]<br>Indinavir<br>Itraconazole<br>Ketoconazole<br>Lopinavir/ Ritonavir[3]<br>Nefazodone<br>Nelfinavir<br>Ritonavir<br>Saquinavir<br>Telithromycin<br>Tipranavir/ Ritonavir[3]	Significant increase in colchicine plasma levels[1]; fatal colchicine toxicity has been reported with clarithromycin, a strong CYP3A4 inhibitor. Similarly, significant increase in colchicines plasma levels is anticipated with other strong CYP3A4 inhibitors.	0.6 mg twice a day<br>0.6 mg once a day	0.3 mg once a day<br>0.3 mg once every other day	1.2 mg (2 tablets) followed by 0.6 mg (1 tablet) 1 hour later. Dose to be repeated no earlier than 3 days.	0.6 mg (1 tablet) x 1 dose, followed by 0.3 mg (1/2 tablet) 1 hour later. Dose to be repeated no earlier than 3 days.	Maximum daily dose of 1.2 – 2.4 mg	Maximum daily dose of 0.6 mg (may be given as 0.3 mg twice a day)
**Moderate CYP3A4 Inhibitors**							
Amprenavir[3]<br>Aprepitant<br>Diltiazem<br>Erythromycin<br>Fluconazole<br>Fosamprenavir[3] (pro-drug of Amprenavir)<br>Grapefruit Juice<br>Verapamil	Significant increase in colchicine plasma concentration is anticipated. Neuromuscular toxicity has been reported with diltiazem and verapamil interactions.	0.6 mg twice a day<br>0.6 mg once a day	0.3 mg twice a day or 0.6 mg once a day<br>0.3 mg once every other day	1.2 mg (2 tablets) followed by 0.6 mg (1 tablet) 1 hour later. Dose to be repeated no earlier than 3 days.	1.2 mg (2 tablets) x 1 dose. Dose to be repeated no earlier than 3 days.	Maximum daily dose of 1.2 – 2.4 mg.	Maximum daily dose of 1.2 mg (may be given as 0.6 mg twice a day)
**P-gp Inhibitors[2]**							
Cyclosporine<br>Ranolazine	Significant increase in colchicine plasma levels[1]; fatal colchicine toxicity has been reported with cyclosporine, a P-gp inhibitor. Similarly, significant increase in colchicine plasma levels is anticipated with other P-gp inhibitors.	0.6 mg twice a day<br>0.6 mg once a day	0.3 mg once a day<br>0.3 mg once every other day	1.2 mg (2 tablets) followed by 0.6 mg (1 tablet) 1 hour later. Dose to be repeated no earlier than 3 days.	0.6 mg (1 tablet) x 1 dose. Dose to be repeated no earlier than 3 days	Maximum daily dose of 1.2 – 2.4 mg	Maximum daily dose of 0.6 mg (may be given as 0.3 mg twice a day)

[1] For magnitude of effect on colchicine plasma concentrations
[2] Patients with renal or hepatic impairment should not be given colchicine in conjunction with strong CYP3A4 or P-gp inhibitors
[3] When used in combination with Ritonavir, see dosing recommendations for strong CYP3A4 inhibitors

Treatment of gout flares with pms-COLCHICINE is not recommended in patients receiving prophylactic dose of Colchicine and CYP3A4 inhibitors.

For patients with severe renal or hepatic impairment, a 3-tablet course is recommended. For these patients, wait at least two weeks before repeating the course (see Warnings and Drug Interactions).

Administration:

- Administer orally with water and maintain adequate fluid intake.
- May be administered without regard to meals.
- May need to supplement with Vitamin B_{12}.
- Avoid grapefruit juice.
- pms-COLCHICINE is not an analgesic medication and should not be used to treat pain from other causes.

OVERDOSE

There is usually a latent period between overdosage and the onset of symptoms, regardless of the route of administration. Deaths have been reported with as little as 7 mg, although higher doses have been taken without fatal results.

The exact dose of colchicine that produces significant toxicity is unknown. A review of 150 patients who overdosed on colchicine found that those who ingested less than 0.5 mg/kg survived and tended to have milder toxicities, such as gastrointestinal symptoms, whereas those who took 0.5 to 0.8 mg/kg had more severe reactions, such as myelosuppression. There was 100% mortality in those who ingested more than 0.8 mg/kg.

Symptoms: The first stage of acute colchicine toxicity typically begins within 24 hours of ingestion and includes gastrointestinal symptoms, such as abdominal pain, nausea, vomiting, diarrhea, and significant fluid loss, leading to volume depletion. Peripheral leukocytosis may also be seen. Life threatening complications occur during the second stage, which occurs 24 to 72 hours after drug administration, attributed to multi-organ failure and its consequences. Death is usually a result of respiratory depression and cardiovascular collapse. If the patient survives, recovery of multi-organ injury may be accompanied by rebound leukocytosis and alopecia starting about 1 week after the initial ingestion.

Treatment: Induce emesis or perform gastric lavage. Symptomatic and supportive treatment. No specific antidote is known. Colchicine is not effectively removed by dialysis.

For management of a suspected drug overdose, contact your regional Poison Control Center immediately.

SUPPLIED

Each yellow, round, biconvex tablet debossed with “C” above “0.6” on one side and scored on the other side contains: 0.6 mg of Colchicine and the following non-medicinal ingredients: D&C Yellow #10, FD&C Yellow #6, magnesium stearate, povidone, sodium starch glycolate and sucrose. Available in HDPE bottle of 100 tablets and in blisters of 30 tablets.

Store at room temperature between 15°C and 30°C.

Keep out of reach and sight of children.

PHARMASCIENCE INC.
6111 Royalmount Ave., Suite 100
Montreal, Quebec, H4P 2T4
www.pharmascience.com

REFERENCES

Colchicine Prescribing Information, Odan Laboratories Ltd, Canada. Date of revision May 4, 2016. Control number: 190129

Appendix C Administrative Procedures for the Reporting of Adverse Events

The following administrative procedures for reporting AEs are to be followed during the conduct of this clinical trial.

1 ADVERSE EVENTS DURING THE TRIAL

Each patient will be observed and queried in a non-specific manner by the investigator or study coordinator at each visit for any new or continuing AE since the previous visit. Any AEs prior to the first dose of study medication will be recorded in the medical history and kept in the patients' chart. All SAEs will be recorded in the appropriate eCRF section. In addition to SAEs, the only AEs to be recorded in the eCRF are those that are either related to the gastrointestinal system, that are judged related to the study medication by the investigator or that are laboratory abnormalities judged clinically significant by the investigator. Information collected will include the onset, duration, severity, relationship to study drug, and the management. SAEs are to be reported if they are known to occur within 30 days following the last study visit.

The investigator will review the clinical laboratory test results in a timely fashion when received from the laboratory. Those results qualifying as AEs as defined in this appendix will be recorded on the AE eCRF section and will be handled according to these AE reporting procedures.

The investigator will review concomitant medications being taken by the patient.

Definitions

1.1 Pre-existing condition

A pre-existing condition is one that is present prior to randomization. A worsening of a pre-existing condition after taking the first dose of investigational product should be reported as an AE.

1.2 Adverse Event (AE)

An AE is defined as any unfavorable and unintended sign (including a clinically meaningful abnormal laboratory finding), symptom, or disease temporally associated with the use of an investigational product, whether or not related to the investigational product.

A medical procedure is not considered and should not be reported as an AE. However, the medical condition which led to the procedure should be considered as an AE and be reported as such.

1.3 Related Adverse Event

A related AE is one where, according to the Investigator, there is a reasonable possibility that the event may have been caused by the study drug.

1.4 Serious Adverse Event (SAE)

Serious Adverse Events (SAE) are those that meet any of the following International Council for Harmonisation (ICH) criteria:

- Is fatal or immediately life-threatening (NOTE: the term "Life-Threatening" refers to an event in which the patient was at immediate risk of death at the time of the event; it does not refer to an event which could hypothetically have caused death had it been more severe);
- Results in persistent or significant disability/incapacity;
- Requires or prolongs patient hospitalization;
- Is a congenital anomaly/birth defect in the offspring of the patient;
- Is a cancer;
- Is an overdose (intentional or accidental);
- Is judged to be medically important.

Medically important events may not be immediately life-threatening, result in death or hospitalization, but may jeopardize the patient or may require intervention to prevent one of the outcomes listed in the definition above. Serious adverse events are to be reported if they are known to occur within 30 days following the last study visit.

Medical and scientific judgment should be exercised in deciding whether other AEs meet these criteria and are immediately reportable to the sponsor or its designee.

Hospitalization is defined as a patient admission to a hospital for medical treatment or observation; a visit to the emergency room for an outpatient consultation is not considered a hospitalization. Moreover, the following hospitalizations are not considered SAEs:

- Hospitalizations for diagnostic or elective surgical procedures for a pre-existing condition.
- Hospitalization for therapy of the target disease(s) of the study if the protocol explicitly anticipated and defined the symptoms or episodes.
- Hospitalization for study efficacy measurement, as defined in the protocol.

1.5 Life-Threatening Adverse Event

A life-threatening AE is an AE that, in the opinion of the investigator, places the patient at immediate risk of death from the reaction as it occurred.

1.6 Unexpected Adverse Event

An unexpected AE is any AE that is not consistent with findings previously observed and described in the current Investigational Medicinal Product Information (IMPI).

1.7 Clinical Laboratory Adverse Event

A clinical laboratory abnormality is regarded as an AE if it has been confirmed by at least 1 repeat test and suggests a disease and/or organ toxicity severe enough to require active management.

1.8 Treatment-Emergent Signs and Symptoms (TESS)

A TESS event is any AE that was not present prior to randomization or that worsens in character, intensity or frequency while the patient is in an active treatment period.

1.9 Post-treatment Adverse Event

A post-treatment AE is any AE that occurs after treatment is discontinued.

2 HANDLING OF ADVERSE EVENTS

2.1 Treatment-Emergent Signs and Symptoms

Any condition/diagnosis that meets the definition of a TESS event is captured in the medical history and kept in the patient's chart.

2.2 Serious Adverse Events

All SAEs are to be immediately reported as outlined in Section 5.2.1.1., Serious Adverse Events, within 24 hours of the Investigator's first knowledge of the event.

2.3 Intensity

The following criteria are used to assess the intensity of each AE:

- Mild: The patient is aware of the sign or symptom, but finds it easily tolerated.
- Moderate: The patient has enough discomfort to cause interference with or change in usual activities.
- Severe: The patient is incapacitated and unable to work or participate in many or all usual activities.

2.4 Relationship to Study Drug – Physician's Assessment

There are 3 categories for the physician's assessment of the causal relationship between study drug and an AE as follows: not related, possibly and probably.

2.5 Clinical Outcome

The following categories are used to assess the clinical outcome of each AE:

- Recovered – The patient has fully recovered from the AE with or without observable residual effects.
- Not Yet Recovered – the patient is still being treated for the residual effects of the original AE. This does not include treatment for pre-existing conditions including the indication for the study drug.
- Died Due to this Adverse Event
- Died, Other Causes
- Unknown
- Surgery/Procedure

3 CAPTURING AVDERSE EVENTS

3.1 Pre-Existing Condition

A pre-existing condition should be captured in the medical history and kept in the patient's chart. If the frequency, intensity, or character of the condition worsens during study treatment, and is either related to the gastrointestinal system, is judged related to the study medication by the investigator or is a laboratory abnormality judged clinically significant by the investigator, it must be documented in the appropriate eCRF AE form.

3.2 Lack of Efficacy of Study Medication

Signs or symptoms defined in the protocol as lack of efficacy or collected as efficacy parameters (endpoints) will be captured as AEs.

3.3 Clinical Laboratory Adverse Event

A clinical laboratory abnormality should be reported as an AE only if it is considered to be clinically significant by the investigator and confirmed by repeat testing.

3.4 Hospitalization or Surgery/Procedure

Any AE reported as study endpoint should not be reported as an SAE. Any condition/diagnosis responsible for surgery/procedure should be reported as an AE if it meets the criteria for an AE. A medical procedure is not considered and should not be reported as an AE. The surgery/procedure itself will be reported as a Clinical Outcome of the underlying event. Events that prolong any hospitalization are reported as SAEs.

3.5 Death

The cause of death should be reported as an AE.

4 REPORTING TO THE SPONSOR

4.1 Immediately Reportable Adverse Events

If an AE meets the definition of Serious, it must be reported IMMEDIATELY in the eCRF. If the eCRF is not available, the investigator should send the paper SAE form by fax (+1-514-461-1301) as soon as possible and within 24 hours of knowledge of the event. Upon return of the availability of the eCRF, the information written on the paper SAE form must be recorded in the eCRF. For any questions or concerns, the investigator may call the MHICC Medical Monitor at +1-514-207-1365 (24/7). If any SAE occurs the investigator, at his discretion, can withdraw the patient from the study while taking the appropriate follow-up action.

MHICC Medical Monitor or his designated representative is responsible to report SUSARs to Regulatory Authorities and to notify Pharmascience Inc.

4.2 Other Adverse Events

Study endpoints are to be reported in the eCRF within 24 hours of awareness of the event.

In addition to SAEs and study endpoints, the only AEs to be recorded on the eCRF are those that are either related to the gastrointestinal system, that are judged related to the study medication by the investigator or that are laboratory abnormalities judged clinically significant by the investigator. Information collected will include the onset, duration, severity, relationship to study drug, and the management.

4.3 Follow-Up Period

For SAEs, the patient must remain under observation until the SAE has subsided or stabilized and all serious findings have returned to normal or stabilized. Any follow-up information to an initial SAE report must be updated in the eCRF. Serious Adverse Events are to be reported if they are known to occur within 30 days following the last study visit.

Appendix D **Other Administrative and Regulatory Procedures**

This appendix provides information necessary to administer the study in compliance with global Good Clinical Practice (GCP) and government regulations.

Your signature on this cover page of the protocol, subsequent amendments, addenda, and the Clinical Trial Agreement confirms that:

- You have been given appropriate information on the study drug
- You have read and understand the protocol and appendices
- You agree to conduct the study in accordance with the provisions of the protocol and applicable regulations
- You acknowledge the sponsor's ownership of the data and results obtained from the conduct of the protocol
- You agree to maintain the confidentiality of information as outlined in this protocol

1. ADMINISTRATIVE PROCEDURES

1.1 Ethics and Informed Consent

1.1.1 Declaration of Helsinki

This study will be conducted in accordance with the Declaration of Helsinki.

1.1.2 Ethics Committee (EC) Review and Approval of the Study

An EC that is organized and operates according to GCP and applicable laws and regulations, should safeguard the rights, safety, and well-being of all trial patients. No patient should be admitted to a trial before the EC issues its written approval/favorable opinion of the trial.

The investigator is responsible for:

- Promptly reporting to the EC all changes in the research activity, all unlabeled AEs, and all unanticipated problems involving risks to human patients or others;
- Not making any changes in the research without EC approval, except when absolutely necessary to eliminate apparent immediate hazards to human patients;
- Submitting a progress report describing the status of the clinical investigation to the EC at appropriate intervals not exceeding 1 year; and
- Submitting a final report when required by the EC within 3 months following completion, termination or discontinuation of the study. Copies of these reports will also be provided **to the sponsor or the sponsor's designated representative**.

In general, all communications with the EC regarding the study will be handled by the principal investigator or coordinating investigator, (if applicable) of the study. The sponsor or the sponsor's designated representative may directly contact the EC if necessary, but must not

attempt to influence the EC in any way. A copy of all communications from the EC to the investigator regarding its review of an initial approval of the study and its re-approvals at intervals must be provided to the sponsor by the investigator.

1.1.3 Informed Consent Form (ICF)

The investigator must fully explain the purpose of the study to the patient or his/her guardian prior to entering the patient into the study. The investigator is responsible for obtaining written informed consent from each patient.

The sponsor requires that the informed consent be obtained orally and on a written form prepared by the investigator and approved by the EC. The person signing the consent form will receive a copy of the signed form. The signed consent form will be filed at the site with the investigator's study files.

The consent form is a written document that includes the informed consent requirements listed below. This form may be read to the patient who will afterward have adequate opportunity to read it, ask questions, and receive requested information before signing.

1.2 Emergency Information

1.2.1 Patient Emergency Information

At least 1 of the investigators at each site will be available to the patient at all times during their participation in the study. The patient will be provided an emergency information card that contains all necessary information to contact the investigator in the case of an emergency, along with a description of the study medications the patient may have received and possible emergency precautions to be taken (if any). The patient will be instructed to carry this card with him/her at all times during the trial.

1.2.2 Emergency Code Breaks

The investigator will be provided with a mechanism for emergency determination of a patient's treatment regimen (if required for proper treatment of the patient) in the event that contact with MHICC is not possible.

1.2.3 Confidentiality of Patient Information

All patients will be assigned a patient number. Subsequently, patients will be identified in the eCRF only by their initials (or coded initials if mandated per local requirements) and that number. Any information published as a result of the study will be such that it will not permit identification of any patient. The information from this study will be available within the sponsor organization and may be shared with the regulatory authorities. It may also be the subject of an audit by a regulatory agency within the local government. The patient's identity will remain protected except as required for legal or regulatory inquiries.

1.2.4 Publication of Study Results

All information and data regarding the study drug obtained in connection with the conduct of this study are considered confidential. Accordingly, the sponsor (Montreal Heart Institute) retains the right to review manuscripts, abstracts, and presentation material related to this protocol and its amendments/addenda prior to presentation or submission to a journal. This review will not restrict publication of facts or opinions formulated by the investigator.

1.2.5 Roles and Responsibilities

Montreal Heart Institute (MHI)
The Montreal Heart Institute is the sponsor for this study.

Montreal Health Innovations Coordinating Center (MHICC)
The MHICC is a division of the Montreal Heart Institute, and has been designated to provide clinical coordination for this trial. These activities include overall project management, site selection, distribution of study materials, site management including clinical monitoring, SUSARs reporting to regulatory authorities, data management, biostatistical analysis and writing of the final clinical study report.

Pharmascience Inc.
Pharmascience Inc. will provide the Montreal Heart Institute with study medication and matched placebo for all patients for the duration of this trial according to a Clinical Supply Agreement between them and MHI (the Sponsor).

Investigator
The Investigator is responsible for ensuring this trial is conducted according to the signed investigator statement, following ICH/GCP guidelines and all other local regulatory requirements; for protecting the rights, safety, and welfare of subjects under the investigator's care; and ensure accountability of the investigational product.

It is the responsibility of the investigator(s) that:

- The study is conducted in accordance with the Declaration of Helsinki and according to the guidelines in the attached appendices.
- This study is conducted in compliance with all applicable laws and regulations of the local and country where the study is conducted.
- This study is not initiated until the protocol and a copy of the informed and consent form (ICF) have been reviewed and approved by a duly constituted Ethics Committee (EC), and that any local institutional requirements are satisfied.
- Each patient and/or their legal guardian (or caregiver) reads, understands, and signs an instrument of informed consent.

- The patient be informed that personal information may be examined during audit by properly authorized individuals but that personal information will be treated as strictly confidential and not be publicly available.
- The patient log and patient records are retained as detailed in this protocol.

The final responsibility for the content of the informed consent statement remains with the investigator and the EC. Indemnification of the investigator, coworkers, and the institution is provided as specified in the Clinical Trial Agreement.

2. PROTOCOL AMENDMENTS AND ADDENDA

2.1 Definitions

A protocol amendment is any systematic change (e.g., revision, addition, deletion) that is made to the Final Protocol for all sites participating in a clinical study and is identified by consecutive Arabic numerals (e.g., Amendment 1, Amendment 2, etc). Amendments can be made regardless of whether the protocol has been signed by the investigator or whether or not the protocol has been implemented at a site.

A Protocol Addendum is any systematic change (e.g., revision, addition, deletion) that is made to the Final Protocol for one/some site(s) but not all sites from a multicenter clinical study and is identified by single sequentially ordered letters (e.g., Addendum A, Addendum B).

An Urgent Protocol Amendment is one that must be instituted quickly, usually to eliminate an apparent immediate hazard to subjects and may be implemented prior to eventual EC review (within 5 working days) and submission to regulatory authorities.

All amendments/addenda to the protocol must be approved by the principal investigator, the sponsor and the EC of the investigator's institution. The investigator is responsible for submitting any proposed change in the approved protocol in writing to the EC for review and approval and for sending a copy of the approval to the sponsor or designee. All amendments/addenda will be filed with appropriate local regulatory authorities by the sponsor or designee.

With the exception of urgent protocol amendments, as outlined below, the amendment/addendum will apply to all subjects/patients entered into the study (or all subjects/patients in affected sites for addenda) after it has gone through the applicable procedure described above and been approved by the EC. Any amendments/addenda proposed in a multicenter protocol must be approved by the EC at the individual study site before it can be placed in effect at that site.

2.2 Urgent Protocol Amendment

If the amendment eliminates an apparent immediate safety hazard to the patient (urgent protocol amendment), it may be implemented immediately. The sponsor will promptly notify the

appropriate regulatory authorities of the amendment while the investigator will notify his/her EC of the change in writing within 5 working days of its implementation.

3. STUDY TERMINATION

The study will normally be carried to completion as described in the protocol. However, if in the course of the study a severe adverse reaction or intercurrent illness is noted in any patient, consideration may be given to abrupt termination of the study for this patient. Such a decision may be made by either the principal investigator or by the sponsor, or both. Likewise, the study may be terminated due to ethical/safety issues or at the sponsor's discretion or for regulatory issues.

Appendix E **Declaration of Helsinki**

Adopted by the 18th WMA General Assembly, Helsinki, Finland, June 1964
and amended by the:
29th WMA General Assembly, Tokyo, Japan, October 1975
35th WMA General Assembly, Venice, Italy, October 1983
41st WMA General Assembly, Hong Kong, September 1989
48th WMA General Assembly, Somerset West, Republic of South Africa, October 1996
52nd WMA General Assembly, Edinburgh, Scotland, October 2000
53rd WMA General Assembly, Washington DC, USA, October 2002 (Note of Clarification added)
55th WMA General Assembly, Tokyo, Japan, October 2004 (Note of Clarification added)
59th WMA General Assembly, Seoul, Republic of Korea, October 2008
64th WMA General Assembly, Fortaleza, Brazil, October 2013

Preamble

1. The World Medical Association (WMA) has developed the Declaration of Helsinki as a statement of ethical principles for medical research involving human subjects, including research on identifiable human material and data.

   The Declaration is intended to be read as a whole and each of its constituent paragraphs should be applied with consideration of all other relevant paragraphs.

2. Consistent with the mandate of the WMA, the Declaration is addressed primarily to physicians. The WMA encourages others who are involved in medical research involving human subjects to adopt these principles.

General Principles

3. The Declaration of Geneva of the WMA binds the physician with the words, “The health of my patient will be my first consideration,” and the International Code of Medical Ethics declares that, “A physician shall act in the patient's best interest when providing medical care.”

4. It is the duty of the physician to promote and safeguard the health, well-being and rights of patients, including those who are involved in medical research. The physician's knowledge and conscience are dedicated to the fulfillment of this duty.

5. Medical progress is based on research that ultimately must include studies involving human subjects.

6. The primary purpose of medical research involving human subjects is to understand the causes, development and effects of diseases and improve preventive, diagnostic and

therapeutic interventions (methods, procedures and treatments). Even the best proven interventions must be evaluated continually through research for their safety, effectiveness, efficiency, accessibility and quality.

7. Medical research is subject to ethical standards that promote and ensure respect for all human subjects and protect their health and rights.

8. While the primary purpose of medical research is to generate new knowledge, this goal can never take precedence over the rights and interests of individual research subjects.

9. It is the duty of physicians who are involved in medical research to protect the life, health, dignity, integrity, right to self-determination, privacy, and confidentiality of personal information of research subjects. The responsibility for the protection of research subjects must always rest with the physician or other health care professionals and never with the research subjects, even though they have given consent.

10. Physicians must consider the ethical, legal and regulatory norms and standards for research involving human subjects in their own countries as well as applicable international norms and standards. No national or international ethical, legal or regulatory requirement should reduce or eliminate any of the protections for research subjects set forth in this Declaration.

11. Medical research should be conducted in a manner that minimizes possible harm to the environment.

12. Medical research involving human subjects must be conducted only by individuals with the appropriate ethics and scientific education, training and qualifications. Research on patients or healthy volunteers requires the supervision of a competent and appropriately qualified physician or other health care professional.

13. Groups that are underrepresented in medical research should be provided appropriate access to participation in research.

14. Physicians who combine medical research with medical care should involve their patients in research only to the extent that this is justified by its potential preventive, diagnostic or therapeutic value and if the physician has good reason to believe that participation in the research study will not adversely affect the health of the patients who serve as research subjects.

15. Appropriate compensation and treatment for subjects who are harmed as a result of participating in research must be ensured.

Risks, Burdens and Benefits

16. In medical practice and in medical research, most interventions involve risks and burdens.

    Medical research involving human subjects may only be conducted if the importance of the objective outweighs the risks and burdens to the research subjects.

17. All medical research involving human subjects must be preceded by careful assessment of predictable risks and burdens to the individuals and groups involved in the research in comparison with foreseeable benefits to them and to other individuals or groups affected by the condition under investigation.

    Measures to minimize the risks must be implemented. The risks must be continuously monitored, assessed and documented by the researcher.

18. Physicians may not be involved in a research study involving human subjects unless they are confident that the risks have been adequately assessed and can be satisfactorily managed.

    When the risks are found to outweigh the potential benefits or when there is conclusive proof of definitive outcomes, physicians must assess whether to continue, modify or immediately stop the study.

Vulnerable Groups and Individuals

19. Some groups and individuals are particularly vulnerable and may have an increased likelihood of being wronged or of incurring additional harm.

    All vulnerable groups and individuals should receive specifically considered protection.

20. Medical research with a vulnerable group is only justified if the research is responsive to the health needs or priorities of this group and the research cannot be carried out in a non-vulnerable group. In addition, this group should stand to benefit from the knowledge, practices or interventions that result from the research.

Scientific Requirements and Research Protocols

21. Medical research involving human subjects must conform to generally accepted scientific principles, be based on a thorough knowledge of the scientific literature, other relevant sources of information, and adequate laboratory and, as appropriate, animal experimentation. The welfare of animals used for research must be respected.

22. The design and performance of each research study involving human subjects must be clearly described and justified in a research protocol.
The protocol should contain a statement of the ethical considerations involved and should indicate how the principles in this Declaration have been addressed. The protocol should include information regarding funding, sponsors, institutional affiliations, potential conflicts of interest, incentives for subjects and information regarding provisions for treating and/or compensating subjects who are harmed as a consequence of participation in the research study.

In clinical trials, the protocol must also describe appropriate arrangements for post-trial provisions.

Research Ethics Committees

23. The research protocol must be submitted for consideration, comment, guidance and approval to the concerned research ethics committee before the study begins. This committee must be transparent in its functioning, must be independent of the researcher, the sponsor and any other undue influence and must be duly qualified. It must take into consideration the laws and regulations of the country or countries in which the research is to be performed as well as applicable international norms and standards but these must not be allowed to reduce or eliminate any of the protections for research subjects set forth in this Declaration.

The committee must have the right to monitor ongoing studies. The researcher must provide monitoring information to the committee, especially information about any serious adverse events. No amendment to the protocol may be made without consideration and approval by the committee. After the end of the study, the researchers must submit a final report to the committee containing a summary of the study's findings and conclusions.

Privacy and Confidentiality

24. Every precaution must be taken to protect the privacy of research subjects and the confidentiality of their personal information.

Informed Consent

25. Participation by individuals capable of giving informed consent as subjects in medical research must be voluntary. Although it may be appropriate to consult family members or community leaders, no individual capable of giving informed consent may be enrolled in a research study unless he or she freely agrees.

26. In medical research involving human subjects capable of giving informed consent, each potential subject must be adequately informed of the aims, methods, sources of funding, any possible conflicts of interest, institutional affiliations of the researcher, the

anticipated benefits and potential risks of the study and the discomfort it may entail, post-study provisions and any other relevant aspects of the study. The potential subject must be informed of the right to refuse to participate in the study or to withdraw consent to participate at any time without reprisal. Special attention should be given to the specific information needs of individual potential subjects as well as to the methods used to deliver the information.

After ensuring that the potential subject has understood the information, the physician or another appropriately qualified individual must then seek the potential subject's freely-given informed consent, preferably in writing. If the consent cannot be expressed in writing, the non-written consent must be formally documented and witnessed.

All medical research subjects should be given the option of being informed about the general outcome and results of the study.

27. When seeking informed consent for participation in a research study the physician must be particularly cautious if the potential subject is in a dependent relationship with the physician or may consent under duress. In such situations the informed consent must be sought by an appropriately qualified individual who is completely independent of this relationship.

28. For a potential research subject who is incapable of giving informed consent, the physician must seek informed consent from the legally authorised representative. These individuals must not be included in a research study that has no likelihood of benefit for them unless it is intended to promote the health of the group represented by the potential subject, the research cannot instead be performed with persons capable of providing informed consent, and the research entails only minimal risk and minimal burden.

29. When a potential research subject who is deemed incapable of giving informed consent is able to give assent to decisions about participation in research, the physician must seek that assent in addition to the consent of the legally authorised representative. The potential subject's dissent should be respected.

30. Research involving subjects who are physically or mentally incapable of giving consent, for example, unconscious patients, may be done only if the physical or mental condition that prevents giving informed consent is a necessary characteristic of the research group. In such circumstances the physician must seek informed consent from the legally authorised representative. If no such representative is available and if the research cannot be delayed, the study may proceed without informed consent provided that the specific reasons for involving subjects with a condition that renders them unable to give informed consent have been stated in the research protocol and the study has been approved by a research ethics committee. Consent to remain in the research must be obtained as soon as possible from the subject or a legally authorised representative.

31. The physician must fully inform the patient which aspects of their care are related to the research. The refusal of a patient to participate in a study or the patient's decision to withdraw from the study must never adversely affect the patient-physician relationship.

32. For medical research using identifiable human material or data, such as research on material or data contained in biobanks or similar repositories, physicians must seek informed consent for its collection, storage and/or reuse. There may be exceptional situations where consent would be impossible or impracticable to obtain for such research. In such situations the research may be done only after consideration and approval of a research ethics committee.

Use of Placebo

33. The benefits, risks, burdens and effectiveness of a new intervention must be tested against those of the best proven intervention(s), except in the following circumstances:

Where no proven intervention exists, the use of placebo, or no intervention, is acceptable; or

Where for compelling and scientifically sound methodological reasons the use of any intervention less effective than the best proven one, the use of placebo, or no intervention is necessary to determine the efficacy or safety of an intervention

and the patients who receive any intervention less effective than the best proven one, placebo, or no intervention will not be subject to additional risks of serious or irreversible harm as a result of not receiving the best proven intervention.

Extreme care must be taken to avoid abuse of this option.

Post-Trial Provisions

34. In advance of a clinical trial, sponsors, researchers and host country governments should make provisions for post-trial access for all participants who still need an intervention identified as beneficial in the trial. This information must also be disclosed to participants during the informed consent process.

Research Registration and Publication and Dissemination of Results

35. Every research study involving human subjects must be registered in a publicly accessible database before recruitment of the first subject.

36. Researchers, authors, sponsors, editors and publishers all have ethical obligations with regard to the publication and dissemination of the results of research. Researchers have a duty to make publicly available the results of their research on human subjects and are accountable for the completeness and accuracy of their reports. All parties should adhere

to accepted guidelines for ethical reporting. Negative and inconclusive as well as positive results must be published or otherwise made publicly available. Sources of funding, institutional affiliations and conflicts of interest must be declared in the publication. Reports of research not in accordance with the principles of this Declaration should not be accepted for publication.

Unproven Interventions in Clinical Practice

37. In the treatment of an individual patient, where proven interventions do not exist or other known interventions have been ineffective, the physician, after seeking expert advice, with informed consent from the patient or a legally authorised representative, may use an unproven intervention if in the physician's judgement it offers hope of saving life, re-establishing health or alleviating suffering. This intervention should subsequently be made the object of research, designed to evaluate its safety and efficacy. In all cases, new information must be recorded and, where appropriate, made publicly available.
