# Supplementary material for: Drug-Drug Interactions and the Clinical Tolerability of Colchicine Among Patients With COVID-19: A Secondary Analysis of the COLCORONA Randomized Clinical Trial
Source: JAMA Netw Open. 2024 Sep 6;7(9):e2431309. doi: 10.1001/jamanetworkopen.2024.31309 (PMC11380098; doi:10.1001/jamanetworkopen.2024.31309)
Supplement: Supplement 2. — Statistical Analysis Plan [file jamanetwopen-e2431309-s002.pdf]

Montreal Health Innovations Coordinating Center | MHICC
A Division of the Montreal Heart Institute

STATISTICAL ANALYSIS PLAN

Protocol number: MHIPS-2020-001

COLCHICINE CORONAVIRUS SARS-CoV2 TRIAL

COLCORONA

Date of Final Statistical Analysis Plan: 24-JUN-2020

Amendment No. 1: 25-NOV-2020

Prepared by:
Malorie Chabot-Blanchet, M.Sc.
Biostatistician
Montreal Health Innovations Coordinating Center (MHICC)
5000 Bélanger Street
Montréal, QC H1T 1C8
Phone: 514-461-1300 ext. 4043 / Fax: 514-461-1301

Signature Approval Page

By signing below, I indicate that I have reviewed the Statistical Analysis Plan in its entirety and approve its contents.

Signature: Jean-Claude Tardif   Date: 2020/11/26
Jean-Claude Tardif, MD
Principal Investigator
MHI Research Center

Signature: Lucie Blondeau for   Date: 26NOV2020
Marie-Claude Guertin, Ph.D.
Lead, Biostatistics
MHICC

Revision History

Version	Date (DD-MMM-YYYY)	Author	Summary of Changes
Final	24-JUN-2020	Malorie Chabot-Blanchet	Initial version
Amendment 1	25-NOV-2020	Malorie Chabot-Blanchet	Updated SAP format.<br>Updated MHICC address.<br><br>**Section 1 Introduction**.<br>Update to annotated CRF version.<br><br>**Section 3.1 Intent-To-Treat Population**<br>Change to the identification of subjects for whom medication was delivered.<br>Added subjects suspected to have eligibility issues to be reviewed for inclusion or exclusion from the ITT population.<br><br>**Section 3.2 Safety Population**<br>Removed “Did the subject take the IP every day since the last visit?” is answered ‘Not Applicable (Subject never took any study medication since the last visit)’) at Day 15 and at EOS as a criterion to be excluded from the safety population.<br><br>**Section 4.2 Secondary Efficacy Endpoints**<br>Added that time from randomization to end of COVID-19 symptoms is defined in the 30 days following randomization.<br><br>**Section 4.3 Exploratory Efficacy Endpoints**<br>Added associations between cytokines and treatment effects.<br><br>**Section 5.1 Adverse Events**<br>Clarified derivation of treatment-emergent AE.

Version	Date (DD-MMM-YYYY)	Author	Summary of Changes
			**Section 6.2 Statistical Considerations**<br>Added for the secondary endpoint "Time from randomization to end of COVID-19 symptoms":<br>- Calculation of the delay between the end of symptoms and randomization.<br>- Derivation of time to censoring in case of a delay > 30 days.<br>- Imputation in case of missing event date.<br><br>**Section 6.3.1 Subject Disposition**<br>Added subject disposition tables to be presented for the ITT population.<br><br>**Section 6.3.3 Datasets Analyzed**<br>Removed number of subjects who had their biomarkers sample collected from table and listing.<br><br>**Section 6.3.4 Demographic and Baseline Characteristics**<br>Added imputation for incomplete date of birth.<br><br>**Section 6.4.2 Secondary Analysis**<br>For the secondary endpoint "time from randomization to end of COVID-19 symptoms":<br>- Changed Kaplan-Meier survival curves for Kaplan-Meier failure curves.<br>- Added subjects for whom the date of first symptoms associated to potential COVID-19 is on the same day as the date of randomization to be considered in the analysis and added specifications on how to deal with incomplete date of first symptoms.<br>-Updated derivation of time to event/censoring.<br>-Updated conditions to use the 15 days phone contact data in place of the EOS data.

Version	Date (DD-MMM-YYYY)	Author	Summary of Changes
			**Section 6.4.3 Exploratory Analysis**<br>Added odds ratio to be provided with the analysis with the viral load measure.<br>Added analyses on the primary endpoint with the cytokines.<br><br>**Section 6.4.4 Subgroup Analysis**<br>Added subgroup analyses for the primary endpoint with the genetic variant rs149354567 and the genetic variant rs10811106 as subgroup factors.<br><br>**Section 6.4.5.4 Analyses on compliant subjects**<br>Added sensitivity analyses for the primary endpoint on subjects for whom the treatment duration is at least 50% of the planned duration and on subjects for whom the treatment duration is at least 75% of the planned duration.<br><br>**Section 6.5.1 Treatment Exposure**<br>Updated variables to be presented.<br><br>**Section 7 Summary Tables, Figures and Listings**<br>Updated to reflect the new analyses.

TABLE OF CONTENTS

1 Introduction 7
2 Study Description 7
2.1 Study Design 7
2.2 Study Objectives 7
3 Datasets Analyzed 8
3.1 Intent-To-Treat (ITT) Population 8
3.2 Safety Population 8
4 Efficacy Endpoints 8
4.1 Primary Efficacy Endpoint 8
4.2 Secondary Efficacy Endpoints 8
4.3 Exploratory Efficacy Endpoints 9
5 Safety Parameters 9
5.1 Adverse Events 9
5.2 Serious Adverse Events 10
6 Statistical Methodology 10
6.1 Determination of Sample Size 10
6.2 Statistical Considerations 10
6.2.1 Interim Analysis 11
6.3 Study Subjects 12
6.3.1 Subject Disposition 12
6.3.2 Protocol Deviations 12
6.3.3 Datasets Analyzed 13
6.3.4 Demographic and Baseline Characteristics 13
6.3.5 Prior and Concomitant Medications 13
6.4 Efficacy Analysis 14
6.4.1 Primary Analysis 14
6.4.2 Secondary Analysis 14
6.4.3 Exploratory Analysis 15
6.4.4 Subgroup Analysis 15
6.4.5 Sensitivity Analysis 16
6.4.5.1 Effect of Baseline Characteristics 16
6.4.5.2 Effect of Missing Data 16
6.4.5.3 Incidence excluding event-free subjects who died prior to study completion 16
6.4.5.4 Analyses on compliant subjects 16
6.5 Safety Analysis 17
6.5.1 Treatment Exposure 17
6.5.2 Adverse Events and Serious Adverse Events 17
7 Summary Tables, Figures and Listings 18

List of Abbreviations

**AE**	Adverse Event
**BMI**	Body Mass Index
**CABG**	Coronary Artery Bypass Graft
**CRF**	Case Report Form
**DSMB**	Data and Safety Monitoring Board
**eCRF**	Electronic Case Report Form
**EOS**	End of Study
**IP**	Investigational Product
**ITT**	Intent-To-Treat
**MHICC**	Montreal Health Innovations Coordinating Center
**MI**	Myocardial Infarction
**PCI**	Percutaneous Coronary Intervention
**SAE**	Serious Adverse Event
**SAP**	Statistical Analysis Plan
**TEAE**	Treatment-Emergent Adverse Event
**TESAE**	Treatment-Emergent Serious Adverse Event

1 Introduction

The purpose of this statistical analysis plan (SAP) is to present the statistical methodology that will be used for the final analysis of Montreal Heart Institute protocol MHIPS-2020-001. This plan also provides a description of the tables, figures and listings that will be included in the final statistical report. It is based on the protocol dated 01-APR-2020 and on the annotated case report form (CRF) version 3.2. In case of differences in terms of descriptions or explanations between the SAP and the protocol, the SAP will supersede the protocol. Any deviation to this SAP would be reported in the statistical report.

2 Study Description

2.1 Study Design

This is a randomized, double-blind, placebo-controlled, multi-center study. Following signature of the informed consent form, approximately 6000 subjects meeting all inclusion and no exclusion criteria will be randomized to receive either colchicine or placebo (1:1 allocation ratio) for 30 days (0.5 mg twice daily for the first 3 days and then once daily for the last 27 days). Follow-up phone or video assessments will occur at 15 and 30 days following randomization for evaluation of the occurrence of any trial endpoints or other adverse events.

Details of the study can be found in the last version of the protocol dated 01-APR-2020.

2.2 Study Objectives

The primary objective of this study is to determine whether short-term treatment with colchicine reduces the rate of death and lung complications related to COVID-19. The secondary objective is to

determine the safety of treatment with colchicine in this subject population. The exploratory objective is to evaluate links between soluble and genetic biomarkers, viral load and treatment effects.

3 DATASETS ANALYZED

Subjects who were not eligible for randomization but who have been erroneously randomized into the study will be excluded from all datasets analyzed if they did not take study product.

3.1 Intent-To-Treat (ITT) Population

The ITT population will consist in randomized subjects (a randomization number is recorded on electronic case report form (eCRF) Form “Randomization”) for whom the study medication was delivered (the IP (investigational product) delivery date is not reported “Not Applicable” on eCRF Form “Study Drug”). Subjects whose condition declined between randomization and delivery of the study medication could be excluded from the ITT population. A listing of these subjects will be reviewed by the principal investigator and the clinical team, who will confirm their inclusion or exclusion from the ITT population prior to unblinding. Subjects randomized by error or subjects suspected to have eligibility issues will also be reviewed by the principal investigator, who will confirm inclusion or exclusion from the ITT population prior to unblinding. In the ITT population, subjects are allocated to treatment groups as per the randomization assignment.

3.2 Safety Population

All subjects who took at least one dose of study medication will be included in the safety analysis population. A randomized subject will be excluded from the safety population if the date of first dose of study medication on eCRF Form “Study Drug” and the date of last dose of study medication on eCRF Form “Study Completion” is ‘Not Applicable’. Subjects will be assigned according to the true treatment received for analysis purposes.

4 EFFICACY ENDPOINTS

4.1 Primary Efficacy Endpoint

The primary endpoint is the composite of death or the need for hospitalization due to COVID-19 infection in the first 30 days after randomization. Death and hospitalization due to COVID-19 will be identified from the eCRF Form “Adverse Event” and derived as outcome of AE= Death Related to Adverse Event or AE term= Hospitalization due to covid19 complication.

4.2 Secondary Efficacy Endpoints

The secondary endpoints are the components of the composite primary endpoint (death and hospitalization due to COVID-19), the need for mechanical ventilation in the first 30 days after randomization and the time from randomization to end of COVID-19 symptoms in the first 30 days after randomization in subjects with symptoms at randomization. The need for mechanical ventilation will be identified from the eCRF Form “Adverse Event”. The event (being symptom-free) and the date of last known symptoms will be identified from the eCRF Form “Subject Status EOS” or “Subject Status D15”.

4.3 Exploratory Efficacy Endpoints

Exploratory endpoints include associations between biomarkers, cytokines and viral load (stored samples) and treatment effects.

In consenting subjects, optional nasal swab will be collected at Visit 2 for the assessment of biomarkers, possibly including but not limited to markers of inflammation, and viral load. Therefore, the exploratory endpoints might not be available in all subjects. These data, as well as the cytokines, will come from external databases.

5 Safety Parameters

Drug safety will be assessed by an evaluation of types, frequencies, severities and duration of any reported AEs. Subjects will be monitored for signs and symptoms of drug toxicity.

5.1 Adverse Events

An AE is defined as any unfavorable and unintended sign (including a clinically meaningful abnormal laboratory finding), symptom, or disease temporally associated with the use of an investigational product, whether or not related to the investigational product.

The only AEs to be recorded on the eCRF, in addition to serious adverse events (SAE), are those that are either related to the gastrointestinal system, that are judged related to the study medication by the investigator or that are laboratory abnormalities judged clinically significant by the investigator. Information regarding AEs will be collected from the first dose of study medication (at Visit 2) through and including the last visit. Information collected will include the onset, end date (if applicable), intensity, relationship to study drug, and the management. Information regarding SAEs that occur within 30 days following the last study visit, and reported to the investigational site, will be collected.

In case the intensity of an AE is missing, it will be considered as 'Severe'.

A treatment-emergent AE is an AE which started after the date of the first dose of study medication or on the date of the first dose of study medication but not prior to the delivery of IP (as reported on eCRF Form "Adverse Event"; if the AE started on the same date as the IP delivery date (on eCRF Form "Study Drug")). In case the date of first dose of study medication is unknown, the AE will be assumed to be treatment-emergent if it did not start prior to the IP delivery. In case the day the event started is unknown, the event will be assumed to be treatment-emergent if it started on the same month and year as the date of first dose of study medication (or IP delivery date if the date of first dose is missing) but did not start prior delivery of IP; otherwise the partial AE start date will be compared to the date of first dose of study medication (or IP delivery date if it is missing) and the AE will be classified accordingly.

A related AE is one where, according to the investigator, there is a reasonable possibility that the event may have been caused by the study drug. They will be identified from the eCRF Form "Adverse Event" as AEs that are possibly or probably related to study treatment.

5.2 Serious Adverse Events

SAEs are those that meet any of the following International Council for Harmonisation (ICH) criteria:

- Is fatal or immediately life-threatening;
- Results in persistent or significant disability/incapacity;
- Requires or prolongs inpatient hospitalization;
- Is a congenital anomaly/birth defect in the offspring of the patient;
- Is a cancer;
- Is an overdose (intentional or accidental);
- Is judged to be medically important.

6 STATISTICAL METHODOLOGY

6.1 Determination of Sample Size

A sample size of approximately 6000 randomized subjects with 3000 subjects in each treatment group will be required to detect a 25% risk reduction with colchicine with a power of 80% given a primary endpoint event rate of 7% in the placebo group and a two-sided test at the 0.05 significance level.

The final analysis of the primary endpoint will be conducted at a significance level slightly below the 0.05 level to account for the interim analysis (see section 6.2.1). However, since this will have a negligible impact on power, the sample size calculation was calculated using a significance level of 0.05.

6.2 Statistical Considerations

Data summaries will be presented using descriptive statistics: N, mean, standard deviation, median, Q1, Q3, minimum and maximum will be presented for continuous variables while count and proportion will be presented for categorical variables.

Basic assumptions of the proposed analyses will be verified prior to the analyses. Since efficacy endpoints death, hospitalization due to COVID-19 infection and need for mechanical ventilation are defined in the 30 days following randomization, the delay between an event of interest and randomization will be calculated as date of the event (date of death, admission date or mechanical ventilation start date) – date of randomization (in days), and any event for which the delay is > 30 days will be excluded from the efficacy analysis. In case the date of the event is incomplete (the exact day could be unknown, but the month and year should be known as they are required fields), the following rule will be applied: Missing event date will be replaced by the maximum between the partially incomplete date where the missing day is replaced by 01 and the randomization date. The secondary endpoint "Time from randomization to end of COVID-19 symptoms" is also defined in the 30 days following randomization. For this analysis, the "event" is to be symptom-free and the "event date" is the date of last known symptoms. The delay between the end of symptoms and randomization will be calculated as (date of last known symptoms – date of randomization). If the delay is > 30 days, the subject will be censored on day 31 (i.e. analyzed as being not symptom-free on day 31; day 1 being the day of randomization for this analysis). Missing event date will be replaced by the minimum between the partially incomplete date where the missing day (or day and month if only the year is known) is

replaced by the last day of the month (or last day of the year) and the date of the visit at which the event was reported (EOS visit if the subject is symptom-free at EOS or Day 15 visit if the subject did not report being symptom-free or not symptom-free at EOS).

Because the main focus of this study is on the primary endpoint, no adjustment to control the overall type I error rate for multiple comparison across the methods used to analyze the primary endpoint and the secondary endpoints is planned. The primary analysis will be conducted at a significance level close to 0.05 (to account for the interim analysis). For the other efficacy analyses, point estimates with 95% confidence intervals will be presented.

Missing data will be imputed in a sensitivity analyses on the primary endpoint described in section 6.4.5.2.

Interim analyses for efficacy and for futility are planned during the study. They are described in section 6.2.1.

Statistical analyses will be performed using SAS Version 9.4 or higher.

6.2.1 Interim Analysis

A fully independent Data and Safety Monitoring Board (DSMB) was established and will review unblinded efficacy and safety data. The DSMB will have the option of recommending early study termination because of overwhelming efficacy, early termination for futility, or continuation of the trial as planned. The method of interim efficacy and futility analyses and the rules for early study termination are detailed in the DSMB charter, approved by all board members, and are described in this section.

Three formal interim efficacy analyses on the primary endpoint will be done after approximately 25%, 50% and 75% of the predicted number of primary events (approximately 368 events). That is, three formal interim analyses on the primary endpoint will be done using the Lan-DeMets procedure with the O'Brien-Fleming alpha spending function to determine the significance level (expecting interim analyses to be conducted at the 0.000015, 0.0030 and 0.0183 significant levels respectively, and final analysis to be conducted at the 0.0440 significant level). The exact significance levels that will be used might be slightly different as they will be computed according to the exact number of events/subjects included at the time of the interim analyses. Of note, the plan is to conduct three interim analyses and the number and timing of these interim analyses are not to be changed according to observed treatment effect. The circumstances under which the number and timing of the interim analyses could be modified include higher/slower than expected enrollment rate or external information.

Futility will be considered by the DSMB in a fashion similar to that for efficacy. Based on the interim analyses, futility will be assessed by computing the conditional power and judged at the 15% level.

The results of the interim statistical analyses and the assessment of futility will be considered as guidance by the DSMB which will be considered along with the results for the secondary efficacy outcomes, the safety profile of the treatments, recruitment and the current medical and public health environment. It should be emphasized that the DSMB will consider all available information before

recommending stopping or extending the trial for safety, efficacy or futility. The decision is not to be taken on statistical considerations alone but also considering for public good in the epidemic.

The interim analyses will be performed by the MHICC unblinded study biostatistician not otherwise involved in the study. More details about the interim analyses can be found in the DSMB charter dated 17-APR-2020.

6.3 Study Subjects

6.3.1 Subject Disposition

Number of subjects randomized, number of subjects who did not take any study medication, number of subjects completing the study and reasons for study discontinuation will be summarized by randomized treatment group, for all randomized subjects as well as for the ITT population. This table will also be presented broken down by Country for all randomized subjects and for the ITT population.

A listing of subject disposition will be presented and will include subject ID, date of positive diagnosis for COVID-19, date of informed consent, date of randomization, IP delivery date, randomization group, age at randomization, sex, date of first dose of study medication, date of last dose of study medication, date of completion/discontinuation and subject's status at study completion.

Subject disposition will also be presented with a flow chart reporting the following information: subjects randomized, subjects for whom the study medication was delivered, subjects who took at least one dose of study medication, subjects completing the study and reasons for study discontinuation.

Delays since first symptoms of COVID-19 will be summarized by treatment group, for the ITT population. The number of days between the date of first symptom of COVID-19 and the following dates will be presented in this table: date of positive diagnosis for COVID-19, date of informed consent and date of first dose of study medication. In addition, the number of days between the date of positive diagnosis of COVID-19 and the date of randomization will be presented, as well as how the diagnosis of COVID-19 was established.

Visit type (how was the visit conducted) for the 15 days visit and EOS visit will also be summarized by treatment group, for the ITT population.

Subject status (Are you symptom-free?, Since your last visit have you been diagnosed with pneumonia?, If Yes, required hospitalization?) at day 15 and at EOS will be summarized by treatment group and overall for the ITT population. In addition, if the date of first symptoms associated to potential COVID-19 infection and the date of last known symptoms are available and complete, the number of days with symptoms will be calculated as (Date of last known symptoms - Date of first symptoms associated to potential COVID-19 infection + 1).

6.3.2 Protocol Deviations

Protocol deviations will be summarized overall and by treatment group for the ITT population. A listing will also be provided and will include subject ID, date of informed consent, date of randomization, randomization group and description and date of deviation.

6.3.3 Datasets Analyzed

The number of subjects in the ITT population and the number of subjects in the Safety population will be summarized overall and by treatment group.

A listing of datasets analyzed will be presented and will include subject ID, randomization group, actual treatment received, ITT population (Yes/No), reason for exclusion from the ITT population, Safety population (Yes/No) and reason for exclusion from the Safety population.

6.3.4 Demographic and Baseline Characteristics

Demographic data will be summarized by treatment group and overall for the ITT population. Baseline characteristics (medical and surgical history and physical data) will also be summarized by treatment group and overall for the ITT population.

In case the date of birth is incomplete, the following imputation will be done:

- the partial date will be replaced by the fifteenth day of the month if month and year are known;
- the partial date will be replaced by the first day of July if only the year is known.

6.3.5 Prior and Concomitant Medications

Prior and concomitant medications will be coded with respect to indication and generic name using the WHO drug dictionary (version MAR2018).

Frequency of use of medications at randomization will be presented for the subjects of the ITT population by therapeutic class and preferred term, overall and for each treatment arm.

A medication will be flagged as being ongoing at the time of randomization if:

- Medication start date < randomization date
  AND
- (Medication end date ≥ randomization date OR Medication is ongoing)

In case of missing or incomplete medication start / end dates, the following rules will be applied:

- If 1) the medication start date is completely missing, or 2) only the year is specified, it is the same as the randomization year and randomization did not occur the first day of the year or 3) only the month/year are specified, they are the same as the randomization month/year and randomization did not occur the first day of the month, then the medication will be assumed to have started before randomization.
- If 1) the medication end date is completely missing, or 2) only the year is specified and it is the same as the randomization year or 3) only the month/year are specified and they are the same as the randomization month/year, then the medication will be assumed to have ended after randomization.

- Otherwise, partial medication start / end dates (month/year or year only) will be compared to the randomization date and the medication will be classified accordingly.

Frequency of use of prior and concomitant medications will also be presented for the subjects of the ITT population by therapeutic class and preferred term, overall and for each treatment arm.

6.4 Efficacy Analysis

All efficacy analyses will be conducted on the ITT population.

6.4.1 Primary Analysis

For each treatment group, the incidence of the primary endpoint, the composite of death or hospitalization due to COVID-19 infection in the first 30 days after randomization, will be calculated as the number of subjects who either died or were hospitalized due to COVID-19 infection in the first 30 days after randomization, divided by the number of subjects randomized to that treatment group. The incidence in each treatment group will then be compared using a chi-square test. Because of the interim analyses, this test might be conducted at a slightly lower significance level than 0.05 (ex. 0.0440). The final significance level will be confirmed prior to database lock and unblinding. The odds ratio will also be provided with the confidence interval calculated at the appropriate level. In addition to the incidence of the endpoint, the delay between the occurrence of the event (the first event if more than one event per subject) and randomization will be summarized.

6.4.2 Secondary Analysis

The secondary endpoints death, hospitalization due to COVID-19 infection and need for mechanical ventilation in the first 30 days after randomization, will be analyzed using chi-square tests, similarly to what is described for the primary endpoint. Odds ratios with 95% confidence intervals will be presented. The delay between the occurrence of the event of interest (the first event if more than one event per subject) and randomization will also be summarized. The incidence per treatment group will be calculated as the number of subjects who experienced the event of interest in the first 30 days after randomization divided by the number of subjects randomized to that treatment group. For hospitalization due to COVID-19 infection and need for mechanical ventilation (endpoints not including mortality), this means that subjects who will die without being hospitalized or needing mechanical ventilation will be included in the denominator. However, for each of these two endpoints, a sensitivity analysis will be conducted excluding the event-free subjects who died prior to study completion. This analysis is described in section 6.4.5.3.

The secondary endpoint time from randomization to end of COVID-19 symptoms will be compared between the two treatment groups using a log rank test and Kaplan-Meier failure curves will be presented. Only subjects for whom the date of first symptoms associated to potential COVID-19 infection is before or on the same day as the date of randomization will be considered in this analysis and subjects for whom the date of last known symptoms is before the date of randomization will be excluded. If the date of first symptoms associated to potential COVID-19 infection is incomplete and the day and month of the partial date is before or is the same as the day and month of randomization, the subject will be considered in the analysis. The time to event (being symptom-free in the 30 days

following randomization) will be calculated as (date of last known symptoms – date of randomization +1), where a missing date of last know symptoms is imputed as described in Section 6.2 (Statistical consideration). Subjects who are not symptom-free ('Are you symptom-free' = No) at EOS will be censored and time to censoring will be computed as the minimum between 31 and (date of EOS phone contact – date of randomization +1 day). For subjects who did not do the EOS phone contact, who did not answer "Are you symptom-free?" by Yes or No at EOS or for whom the time to event/censoring cannot be derived, the 15 days phone contact data will be used in place of the EOS data in the above definitions.

6.4.3 Exploratory Analysis

For each biomarker, exploratory analysis will consist in logistic regression models with the primary endpoint and secondary endpoints death, hospitalization due to COVID-19 infection and need for mechanical ventilation in the first 30 days after randomization (yes/no) as dependant variables and treatment group, biomarker and the treatment group x biomarker interaction as independent variables. This interaction term will indicate whether the treatment effect is affected by the level of the biomarker. The odds ratio will be provided with the 95% confidence interval for treatment group comparison given that the biomarker is taking the value of the 1st quartile, median and 3rd quartile of its distribution.

Similar analysis will be conducted with the viral load measure in place of the biomarker in the models. For the analysis with the viral load measure, the odds ratio for a one unit increase, with a 95% confidence interval, will also be provided for each treatment group.

Similar analysis will also be conducted on the primary endpoint, with each of the cytokine in place of the biomarker.

6.4.4 Subgroup Analysis

Subgroup analyses will be performed for the primary endpoint, looking at the homogeneity of the treatment effect across the following factors: age at randomization (≥ 70 years or < 70 years), sex, race (black or not black), body mass index (BMI) at screening (≥ 30 kg/m^2 or < 30 kg/m^2), smoking status at screening, history of diabetes, history of hypertension, history of respiratory disease, history of coronary disease or heart failure (prior myocardial infarction (MI), prior percutaneous coronary intervention (PCI), prior coronary artery bypass graft (CABG) or prior heart failure), use of agent acting on the renin-angiotensin-aldosterone system at randomization and how was the COVID-19 diagnosis established. The subgroup analyses will be conducted using logistic regression models including the treatment group, the subgroup factor and the treatment x subgroup factor interaction. This interaction will be tested at the 0.10 significance level and will determine whether the treatment effect is impacted by the presence of the factor. Treatment effects will also be estimated and presented with 95% confidence interval within subgroups.

Similar subgroup analyses for the primary endpoint will be performed with the genetic variant rs149354567 (genotype: G G, G GA or GA GA) and the genetic variant rs10811106 (genotype: C C, C T or T T) as subgroup factors. For these two analyses, however, the interaction will be tested at the 0.05 significance level. The genetic parameters will come from an external database.

6.4.5 Sensitivity Analysis

6.4.5.1 Effect of Baseline Characteristics

As a sensitivity analysis for the primary endpoint, a multivariable logistic regression model will be used with occurrence of the primary endpoint (yes/no) as the dependent variable and, as potential independent variables, the treatment group (forced in the model) as well as the following important baseline characteristics: age at randomization, sex, BMI at screening, smoking status at screening, history of diabetes, history of hypertension, history of respiratory disease, prior MI, prior coronary revascularization (prior PCI or prior CABG) and prior heart failure. The results from univariable logistic models with only the baseline characteristic as the independent variable will be carefully reviewed and baseline characteristics that will 1) show an association (p-value<0.20) with the primary endpoint, 2) not have levels with too few subjects and 3) not show too many missing values will be candidate for inclusion in a stepwise multivariable logistic regression model in which the treatment group will be forced. Adjusted odds ratios along with 95% confidence intervals will be provided.

6.4.5.2 Effect of Missing Data

Because of the short follow-up (30 days) and the nature of the primary endpoint (death or hospitalization), no missing primary endpoint data is expected. In other words, for all randomized subjects, it is expected that the information on death or hospitalization during the follow-up will be retrieved. However, should there be any subjects for whom no death or hospitalization due to COVID-19 infection is reported in the eCRF (event-free subjects) and who discontinued the study prior to day 30 or for whom no information is available at the EOS phone call (visit not done or Were any new adverse events experienced? = Unknown), a sensitivity analysis will be conducted in which these subjects will be assumed to have experienced a primary event in the 30 days following randomization. The primary analysis described in section 6.4.1 will be repeated with these imputed events.

6.4.5.3 Incidence excluding event-free subjects who died prior to study completion

As sensitivity analysis for hospitalization due to COVID-19 infection and need for mechanical ventilation (endpoints not including mortality), the secondary analysis of section 6.4.2 will be repeated with the incidence per treatment group calculated as the number of subjects who experienced the event of interest in the first 30 days after randomization divided by the number of subjects randomized to that group and who either had the event of interest or were event-free and did not discontinue the study due to death.

6.4.5.4 Analyses on compliant subjects

As sensitivity analyses for the primary endpoint, the primary analysis will be repeated on subjects for whom the treatment duration is at least 50% of the planned duration (planned duration: 30 days) and on subjects for whom the treatment duration is at least 75% of the planned duration; treatment duration being used as a measure of compliance. Treatment duration will be calculated as (date of last dose of study medication – date of first dose of study medication +1). In case the date of last dose is missing, the study completion date will be used instead. In case the date of first dose is missing, the date

of delivery of IP will be used instead. If the date of delivery of IP is also missing, it will be replaced by the date of randomization.

Subjects with a treatment duration ≥ 15 days will be included in the first analysis (duration is at least 50%) and subjects with a treatment duration ≥ 23 days will be included in the second analysis (duration is at least 75%).

6.5 Safety Analysis

The safety analyses described in this section will be conducted on the safety population. No formal statistical testing is planned for the safety parameters.

6.5.1 Treatment Exposure

Exposure to study medication at Day 15 and at EOS (did subject take the IP every day since the last visit (yes/no/not applicable) and number of days the study medication has been missed since last visit) will be summarized by treatment group, for the safety population. Number of days in the study (calculated as (Date of study completion/discontinuation – Date of randomization +1)), treatment duration (calculation described above in section 6.4.5.4) and number of pills taken (calculated as (35- number of pills left in the bottle (as entered on eCRF Form “Compliance”)) will also be presented with descriptive statistics.

6.5.2 Adverse Events and Serious Adverse Events

AEs will be coded by system organ class and preferred term according to the MedDRA dictionary (version 22.1).

Treatment-emergent AEs (TEAE) will be summarized. Number and proportion of subjects experiencing a treatment-emergent AE will be presented by system organ class, preferred term and intensity for each treatment arm.

The total number of TEAEs reported, the number and proportion of subjects experiencing at least one TEAE, at least one severe TEAE, at least one TEAE related to the study treatment, and at least one TEAE leading to drug withdrawal will also be presented.

Because in this study adverse events are either categorized as SAE, gastrointestinal AE, AE related to study drug as per investigator judgment or laboratory abnormality, the presentation described above will be done for each category of AE.

All SAEs will also be listed. The listing will include subject ID, randomization group, age at randomization, sex, date of first dose of study medication, name and preferred term of AE, SOC term, start and end date (if applicable), type of SAE, treatment/procedure performed for the event, intensity, relationship to study drug, action taken and outcome.

A separate listing for deaths will be also provided. This listing will include subject ID, randomization group, age at randomization, sex, date of first dose of study medication, date of death as well as the cause of death and the AE term leading to death.

7 SUMMARY TABLES, FIGURES AND LISTINGS

1 Study subject	
1.1 Subject disposition	
Table 1.1.1	*Subject disposition*
Table 1.1.1b	*Subject disposition, ITT population*
Table 1.1.2	*Subject disposition by country the subject is treated in*
Table 1.1.2b	*Subject disposition by country the subject is treated in, ITT population*
Table 1.1.3	*Delays since first symptoms of COVID-19 and positive diagnosis, ITT population*
Table 1.1.4	*Visit information, ITT population*
Table 1.1.5	*Summary of subject status at day 15, ITT population*
Table 1.1.6	*Summary of subject status at EOS, ITT population*
Table 1.1.7	*Protocol deviations, ITT population*
Table 1.1.8	*Datasets analyzed*
1.2 Demographic and baseline characteristics	
Table 1.2.1	*Summary of demographics, ITT population*
Table 1.2.2	*Summary of medical and surgical history, ITT population*
Table 1.2.3	*Summary of physical data, ITT population*
1.3 Prior and concomitant medication	
Table 1.3.1	*Summary of medication at randomization, ITT population*
Table 1.3.2	*Summary of prior and concomitant medication, ITT population*

2 Efficacy analysis	
2.1 Primary analysis	
Table 2.1.1	*Summary of and statistical analysis for composite of death or hospitalization due to COVID-19 infection, ITT population*
2.2 Secondary analysis	
Table 2.2.1	*Summary of and statistical analysis for death, ITT population*
Table 2.2.2	*Summary of and statistical analysis for hospitalization due to COVID-19 infection, ITT population*
Table 2.2.3	*Summary of and statistical analysis for need for mechanical ventilation, ITT population*
Table 2.2.4	*Summary of and statistical analysis for time from randomization to end of COVID-19 symptoms, ITT population*
2.3 Exploratory analysis	
Table 2.3.1	*Summary of and statistical analysis for impact of biomarker 1 on composite of death or hospitalization due to COVID-19 infection, ITT population*
Table 2.3.2	*Summary of and statistical analysis for impact of biomarker 1 on death, ITT population*
Table 2.3.3	*Summary of and statistical analysis for impact of biomarker 1 on*

	hospitalization due to COVID-19 infection, ITT population
Table 2.3.4	*Summary of and statistical analysis for impact of biomarker 1 on need for mechanical ventilation, ITT population*
Each biomarker and viral load parameter will be presented in separate tables similarly as tables 2.3.1, 2.3.2, 2.3.3 and 2.3.4.Each cytokine will be presented in separate tables similarly as table 2.3.1.	
2.4 Subgroup analysis	
Table 2.4.1	*Composite of death or hospitalization due to COVID-19 infection broken down by age at randomization (≥ 70 years or < 70 years), ITT population*
Table 2.4.2	*Composite of death or hospitalization due to COVID-19 infection broken down by sex, ITT population*
Table 2.4.3	*Composite of death or hospitalization due to COVID-19 infection broken down by race (black or not black), ITT population*
Table 2.4.4	*Composite of death or hospitalization due to COVID-19 infection broken down by BMI at screening (≥ 30 kg/m^2 or < 30 kg/m^2), ITT population*
Table 2.4.5	*Composite of death or hospitalization due to COVID-19 infection broken down by smoking status at screening, ITT population*
Table 2.4.6	*Composite of death or hospitalization due to COVID-19 infection broken down by history of diabetes, ITT population*
Table 2.4.7	*Composite of death or hospitalization due to COVID-19 infection broken down by history of hypertension, ITT population*
Table 2.4.8	*Composite of death or hospitalization due to COVID-19 infection broken down by history of respiratory disease, ITT population*
Table 2.4.9	*Composite of death or hospitalization due to COVID-19 infection broken down by history of coronary disease or heart failure, ITT population*
Table 2.4.10	*Composite of death or hospitalization due to COVID-19 infection broken down by use of agent acting on the renin-angiotensin-aldosterone system at randomization, ITT population*
Table 2.4.11	*Composite of death or hospitalization due to COVID-19 infection broken down by how was the COVID-19 diagnosis established, ITT population*
Table 2.4.12	*Composite of death or hospitalization due to COVID-19 infection broken down by genotypes for genetic variant rs149354567, ITT population*
Table 2.4.13	*Composite of death or hospitalization due to COVID-19 infection broken down by genotypes for genetic variant rs10811106, ITT population*
2.5 Sensitivity analysis	
Table 2.5.1	*Summary of and stepwise multivariable logistic regression model for composite of death or hospitalization due to COVID-19 infection, ITT population*
Table 2.5.2	*Summary of and univariable logistic regression model for composite of death or hospitalization due to COVID-19 infection ,variable: age at randomization , ITT population*
Table 2.5.3	*Summary of and univariable logistic regression model for composite of death or hospitalization due to COVID-19 infection ,variable: sex, ITT population*
Table 2.5.4	*Summary of and univariable logistic regression model for composite of death or hospitalization due to COVID-19 infection ,variable: BMI at screening, ITT*

	population
Table 2.5.5	*Summary of and univariable logistic regression model for composite of death or hospitalization due to COVID-19 infection ,variable: smoking status at screening, ITT population*
Table 2.5.6	*Summary of and univariable logistic regression model for composite of death or hospitalization due to COVID-19 infection ,variable: history of diabetes, ITT population*
Table 2.5.7	*Summary of and univariable logistic regression model for composite of death or hospitalization due to COVID-19 infection ,variable: history of hypertension, ITT population*
Table 2.5.8	*Summary of and univariable logistic regression model for composite of death or hospitalization due to COVID-19 infection ,variable: history of respiratory disease, ITT population*
Table 2.5.9	*Summary of and univariable logistic regression model for composite of death or hospitalization due to COVID-19 infection ,variable: prior MI, ITT population*
Table 2.5.10	*Summary of and univariable logistic regression model for composite of death or hospitalization due to COVID-19 infection ,variable: prior coronary revascularization, ITT population*
Table 2.5.11	*Summary of and univariable logistic regression model for composite of death or hospitalization due to COVID-19 infection ,variable: prior heart failure, ITT population*
Table 2.5.12	*Summary of and statistical analysis for composite of death or hospitalization due to COVID-19 infection, with missing data imputation, ITT population*
Table 2.5.13	*Summary of and statistical analysis for hospitalization due to COVID-19 infection, excluding event-free deceased subjects, ITT population*
Table 2.5.14	*Summary of and statistical analysis for the need for mechanical ventilation, excluding event-free deceased subjects, ITT population*
Table 2.5.15	*Summary of and statistical analysis for composite of death or hospitalization due to COVID-19 infection, on subjects with a treatment duration ≥ 15 days, ITT population*
Table 2.5.16	*Summary of and statistical analysis for composite of death or hospitalization due to COVID-19 infection, on subjects with a treatment duration ≥ 23 days, ITT population*

3 Safety analysis	
3.1 Treatment exposure	
Table 3.1.1	*Summary of exposure to study medication, safety population*
3.2 Adverse events and serious adverse events	
Table.3.2.1	*Overall summary of treatment emergent SAEs (TESAE), safety population*
Table 3.2.2	*Summary of treatment emergent SAEs (TESAE) by preferred term, system organ class and intensity, safety population*
Table 3.2.3	*Overall summary of gastrointestinal treatment emergent AEs (TEAE), safety population*
Table 3.2.4	*Summary of gastrointestinal treatment emergent AE (TEAE)s by preferred term, system organ class and intensity, safety population*

Table 3.2.5	*Overall summary of treatment emergent AEs (TEAE) related to study drug, safety population*
Table 3.2.6	*Summary of treatment emergent AEs (TEAE) related to study drug by preferred term, system organ class and intensity, safety population*
Table 3.2.7	*Overall summary of treatment emergent laboratory abnormalities, safety population*
Table 3.2.8	*Summary of treatment emergent laboratory abnormalities by preferred term, system organ class and intensity, safety population*

Figures	
Figure 1	*Subject Disposition (flow chart)*
Figure 2	*Kaplan-Meier failure curves for time from randomization to end of COVID-19 symptoms, ITT population*

Listings	
Listing 1	*Subject Disposition*
Listing 2	*Protocol Deviations*
Listing 3	*Datasets analyzed*
Listing 4	*SAEs*
Listing 5	*Deaths*
