# Supplementary material for: Drug-Drug Interactions and the Clinical Tolerability of Colchicine Among Patients With COVID-19: A Secondary Analysis of the COLCORONA Randomized Clinical Trial
Source: JAMA Netw Open. 2024 Sep 6;7(9):e2431309. doi: 10.1001/jamanetworkopen.2024.31309 (PMC11380098; doi:10.1001/jamanetworkopen.2024.31309)
Supplement: Supplement 3. — eFigure. CONSORT Flow Diagram eTable 1. Comparison of Drug-Drug Interaction Classifications eTable 2. Use of Drugs That Interact With Colchicine at Baseline [file jamanetwopen-e2431309-s003.pdf]

Supplementary Online Content

Alfehaid LS, Farah S, Omer A, et al. Drug-drug interactions and the clinical tolerability of colchicine among patients with COVID-19. *JAMA Netw Open*. 2024;7(9):e2431309. doi:10.1001/jamanetworkopen.2024.31309

eFigure. CONSORT Flow Diagram

eTable 1. Comparison of Drug-Drug Interaction Classifications

eTable 2. Use of Drugs That Interact With Colchicine at Baseline

This supplementary material has been provided by the authors to give readers additional information about their work.

© 2024 Alfehaid LS et al. *JAMA Network Open.*

eFigure. CONSORT flow diagram.

DDI = drug-drug interaction; eGFR = estimated glomerular filtration rate

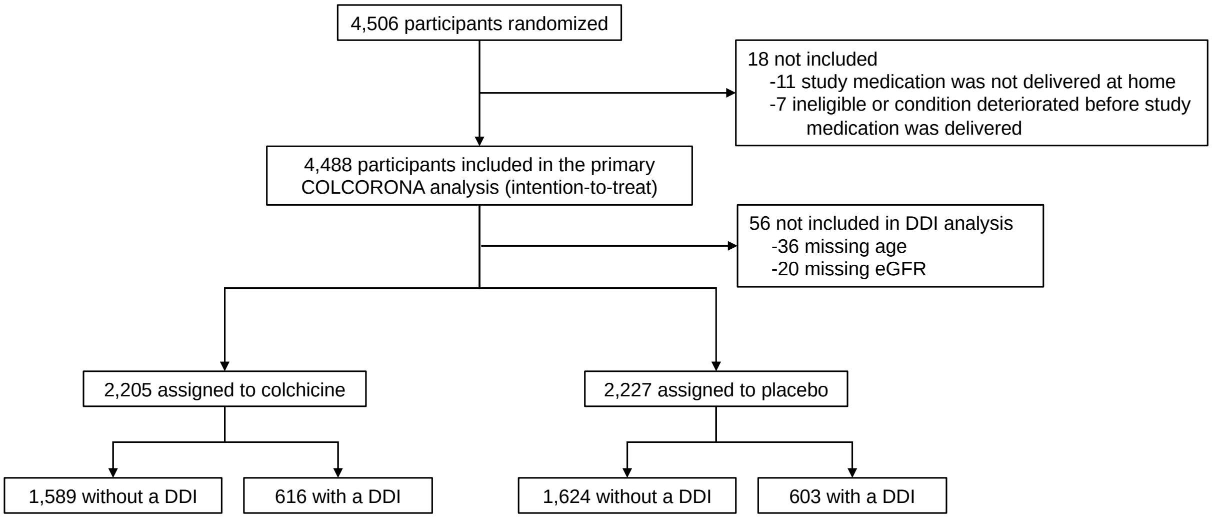

© 2024 Alfehaid LS et al. *JAMA Network Open.*

eTable 1. Comparison of drug-drug interaction classifications

Caption: The ORCA classification system shows good agreement with other drug-drug interaction classification systems. The MicroMedex system classified carvedilol as "Contraindicated" due to inhibition of P-gp with fair evidence grade ("Available documentation is poor, but pharmacologic considerations lead clinicians to suspect the interaction exists; or, documentation is good for a pharmacologically similar drug.") and fluvoxamine as "Major" due to inhibition of CYP3A4 with a fair evidence grade. The LexiComp system categorizes lasmiditan as a Risk Rating X (avoid combination) based on a pharmacokinetic study of lasmiditan and dabigatran.

Drug-Drug Interaction Type	ORCA Class 5 (no interaction)	ORCA Class 1-4
FDA Strong CYP3A4	0	0
FDA Moderate CYP3A4	0	41 (3%)
FDA P-gp Inhibitor	0	2 (<1%)
FDA Other DDI	0	622 (51%)
MicroMedex Contraindicated	11 (<1%)	58 (5%)
MicroMedex Major	3 (<1%)	1,142 (94%)
LexiComp Risk Rating X	1 (<1%)	0
LexiComp Risk Rating D	0	0
LexiComp Risk Rating C	0	1,142 (94%)

© 2024 Alfehaid LS et al. *JAMA Network Open.*

eTable 2. Use of drugs that interact with colchicine at baseline

Drug	ORCA Class	Placebo (n=2227)	Colchicine (n=2205)
Rosuvastatin	4	251 (11%)	282 (13%)
Atorvastatin	3	235 (11%)	226 (10%)
Simvastatin	3	39 (2%)	33 (2%)
Fenofibrate	3	30 (1%)	28 (1%)
Azithromycin	3	29 (1%)	26 (1%)
Diltiazem	2	20 (<1%)	19 (<1%)
Pravastatin	4	19 (<1%)	18 (<1%)
Hydroxychloroquine	4	10 (<1%)	12 (<1%)
Gemfibrozil	3	1 (<1%)	3 (<1%)
Digoxin	4	3 (<1%)	0
Phenobarbital	2	0	3 (<1%)
Tacrolimus	3	2 (<1%)	1 (<1%)
Amiodarone	2	2 (<1%)	0
Ranolazine	3	2 (<1%)	0
Fluvastatin	4	1 (<1%)	0
Chloroquine	4	1 (<1%)	0
Lovastatin	3	0	1 (<1%)
Erythromycin	2	0	1 (<1%)
Fluconazole	3	0	1 (<1%)

© 2024 Alfehaid LS et al. *JAMA Network Open.*
