# Supplementary material for: Drug-Drug Interactions and the Clinical Tolerability of Colchicine Among Patients With COVID-19: A Secondary Analysis of the COLCORONA Randomized Clinical Trial
Source: JAMA Netw Open. 2024 Sep 6;7(9):e2431309. doi: 10.1001/jamanetworkopen.2024.31309 (PMC11380098; doi:10.1001/jamanetworkopen.2024.31309)
Supplement: Supplement 4. — Data Sharing Statement [file jamanetwopen-e2431309-s004.pdf]

Data Sharing Statement

Alfehaid. Open Drug-Drug Interactions and the Clinical Tolerability of Colchicine Among Patients With COVID-19. *JAMA Netw Open*. Published September 05, 2024.
doi:10.1001/jamanetworkopen.2024.31309

Data
Data available: Yes
Data types: Other (please specify)
Additional Information: Interested investigators can apply for data access through Vivli.org.
How to access data: Interested investigators can apply for data access through Vivli.org.
When available: With publication

Supporting Documents
Document types: None

Additional Information
Who can access the data: Interested investigators can apply for data access through Vivli.org.
Types of analyses: Interested investigators can apply for data access through Vivli.org.
Mechanisms of data availability: Interested investigators can apply for data access through Vivli.org.
